# Hypoxic Microenvironment Effects on Matrix Metalloproteinases’ Expression and Enzymatic Activity, and HIFs’ Expression in Non-Small Cell Lung Cancer Cells

**DOI:** 10.3390/ijms27209314

**Published:** 2026-10-20

**Authors:** Georgina Gonzalez-Avila, Cuauhtemoc Sandoval, Bettina Sommer, A. Armando Garcia-Hernandez, Jose S. Lopez-Gonzalez, Arnoldo Aquino-Gálvez, Carina Becerril, Edgar Flores-Soto

**Affiliations:** 1 Departamento de Investigación en Enfermedades Crónico Degenerativas, Instituto Nacional de Enfermedades Respiratorias “Ismael Cosío Villegas”, Calzada de Tlalpan 4502, Col. Sección XVI, Tlalpan, Mexico City 14080, Mexico; tememosan@yahoo.com.mx; 2 Departamento de Investigación en Hiperreactividad Bronquial, Instituto Nacional de Enfermedades Respiratorias “Ismael Cosío Villegas”, Calzada de Tlalpan 4502, Col. Sección XVI, Tlalpan, Mexico City 14080, Mexico; bsommer195@gmail.com; 3 Departamento de Biomedicina Molecular e Investigación Traslacional, Instituto Nacional de Enfermedades Respiratorias “Ismael Cosío Villegas”, Calzada de Tlalpan 4502, Col. Sección XVI, Tlalpan, Mexico City 14080, Mexico; armandgh16@gmail.com; 4 Laboratorio de Cáncer Pulmonar, Instituto Nacional de Enfermedades Respiratorias “Ismael Cosío Villegas”, Calzada de Tlalpan 4502, Col. Sección XVI, Tlalpan, Mexico City 14080, Mexico; slopezgonzalez@yahoo.com; 5 Laboratorio de Biología Molecular, Instituto Nacional de Enfermedades Respiratorias “Ismael Cosío Villegas”, Calzada de Tlalpan 4502, Col. Sección XVI, Tlalpan, Mexico City 14080, Mexico; araquiga@yahoo.com.mx; 6 Laboratorio de Biología Celular, Instituto Nacional de Enfermedades Respiratorias “Ismael Cosío Villegas”, Calzada de Tlalpan 4502, Col. Sección XVI, Tlalpan, Mexico City 14080, Mexico; lcbb6@hotmail.com; 7 Departamento de Farmacología, Facultad de Medicina, Universidad Nacional Autónoma de México, Coyoacán, Mexico City 04510, Mexico; edgarfloressoto@yahoo.com.mx

**Keywords:** HIFs, hypoxia, lung cancer, MMP-2, MMP-9, NSCLC, tumor microenvironment (TME)

## Abstract

The hypoxic tumor microenvironment (TME) promotes metastasis, in which matrix metalloproteinases (MMPs) play a central role. The present work aimed to analyze the effects of hypoxia on MMP expression and enzymatic activity, as well as hypoxia-inducible factor (HIF) expression in different non-small cell lung cancer (NSCLC) cells. Cells were cultured in 1% O_2_ for 6 and 12 h, and the effects of hypoxia on MMPs and HIFs were analyzed using Western blotting and zymography. The highest MMP-2 protein expression was detected in CALU-1 (*p* ≤ 0.003) and A549 cells (*p* ≤ 0.006) at 6 and 12 h, respectively. Higher levels of MMP-9 were detected in CALU-1 (*p* ≤ 0.003) at 6 h and in A549 cells (*p* ≤ 0.04) at 12 h. MMP-2 and MMP-9 had the highest enzymatic activity in SKMES-1 cells (*p* ≤ 0.003). Increased HIF-1α levels were detected in A427 (*p* ≤ 0.04) at 6 h and in SKLU-1 cells (*p* ≤ 0.01) at 12 h, while higher HIF-2α expression was found in SKMES-1 at 6 h and 12 h (*p* ≤ 0.04). In conclusion, although hypoxia induces changes in MMP-2 and MMP-9 expression in NSCLC cells through HIF-dependent or HIF-independent pathways, these effects are not necessarily similar to those observed in enzymatic activity and differ between cell lines.

## 1. Introduction

The International Agency for Research on Cancer (IARC) reported 20.64 million new cases of cancer, with a mortality of 9.76 million subjects in 2024, with lung cancer occupying the first place in incidence and mortality for men, and the second place for women [1]. The leading cause of death among lung cancer patients is metastasis, which transforms a local disease into a systemic one [2].

Lung cancer pathogenesis is related to exposure to external carcinogens, such as tobacco smoke, biomass smoke, and pollution, which, together with DNA mutations caused by DNA repair system mistakes, provoke genetic and epigenetic alterations in lung cells [3,4]. These modified cells gain an adaptive advantage over other cells in the tissue, enabling them to survive in a changing microenvironment and eventually become neoplastic cells. Cancer cells increase their proliferation rate and, consequently, their energy consumption, thereby lowering nutrient and oxygen levels in their microenvironment. Adaptation to an acidic and hypoxic tumor microenvironment (TME) is not uniform across all tumor cells, since it depends on their genetic and epigenetic characteristics [5]. Within this intratumor heterogeneity (ITH), a group of cells acquires the ability to initiate the metastatic process.

Metastasis comprises several stages that enable cancer cells to detach from the primary tumor, migrate through neighboring tissues, and enter a blood or lymphatic vessel, where they circulate until they reach a distant site and form a metastatic colony [6]. Among the molecules that participate in all the steps of the metastatic chain are the matrix metalloproteinases (MMPs) [7]. The MMPs are endopeptidases that require zinc and calcium for their enzymatic activity. Twenty-eight different MMPs have been identified in vertebrates and twenty-four in humans, including MMP-23A and MMP-23B. Based on their substrate specificity and molecular structure, MMPs are classified into gelatinases, collagenases, stromelysins, matrilysins, glycosylphosphatidylinositol (GPI)-anchored MMPs, transmembrane type I MMPs, transmembrane type II MMPs, and other MMPs [7]. MMPs are synthesized as zymogens, except MMP-23, which lacks a pro-peptide domain. MMP expression and activity are upregulated in many cancers and have been associated with poor prognosis. Therefore, identifying molecular mechanisms that enhance their expression and enzymatic activity in the hypoxic TME might uncover novel targets to develop more effective therapeutic strategies.

Likewise, hypoxic signaling is mainly mediated by the hypoxia-inducible factors (HIFs), which regulate the expression of multiple genes, allowing neoplastic cells to adapt to a “hostile” microenvironment [8,9]. Three HIF-α chains have been identified: HIF-1α, HIF-2α (Endothelial PAS domain-containing protein 1, EPAS1), and HIF-3α. Their expression and stabilization are subject to changes in oxygen concentration. Under normoxic conditions, HIF-α chains are hydroxylated and bind to the von Hippel-Lindau protein (pVHL), leading to their ubiquitination and proteasomal degradation [9]. In contrast, under hypoxia, HIF-α chains translocate into the nucleus and bind to the HIF-β chains. The dimers form a complex with the coactivator cAMP-response-element binding protein (CREB)-binding protein (CBP)/p300, which joins to the 5′G/ACGTG3′ hypoxia response element (HRE) present in the promoter site of the target genes.

Because MMPs and HIFs are important in metastasis, this work aimed to identify the effects of a hypoxic TME on MMP expression and enzymatic activity, and on HIF expression, across different NSCLC cell lines.

## 2. Results

### 2.1. MMP-2 Expression

Western blot analysis was performed to assess MMP-2 expression in the culture medium of NSCLC cell lines (Figure 1A). Bands of about 72 kDa (proMMP-2 or latent MMP-2 form) and 65 kDa (active MMP-2 form) were visualized in the media of all lung cancer cell lines (Figure 1A). MMP-2 protein expression densitometry results for each cell line under normoxic and hypoxic conditions at 6 and 12 h are presented below (Figure 1B). Figure 1C compares MMP-2 expression among NSCLC cell lines.

#### 2.1.1. A427 Cell Line MMP-2 Expression

The densitometry analysis showed a significant increase in proMMP-2 protein expression in hypoxic versus normoxic conditions at 6 h (26,650.4 ± 768.5, and 19,596.6 ± 744.8 D.U., respectively; * *p* = 0.003), and 12 h (28,429.7 ± 1050.2, and 19,411.4 ± 580.4 D.U., respectively; ** *p* = 0.02) in A427 cells (Figure 1B). Active MMP-2 was significantly increased under hypoxia at 6 h compared to normoxic conditions (21,237.8 ± 936.7 and 13,643.9 ± 961.4 D.U., respectively; ^†^ *p* = 0.03). No significant differences were observed between normoxic and hypoxic conditions when cells were cultured under hypoxia for 12 h.

#### 2.1.2. A549 Cell Line MMP-2 Expression

No significant differences in proMMP-2 expression were observed between hypoxic and normoxic conditions in A549 cells at 6 and 12 h (Figure 1B). Additionally, active MMP-2 was increased at 12 h under hypoxia relative to normoxia (103,196.4 ± 10,430.6 and 25,135.6 ± 6032.4 D.U., respectively; ^†^ *p* = 0.001). Active MMP-2 levels did not differ significantly between hypoxic and normoxic conditions at 6 h.

#### 2.1.3. SKMES-1 Cell Line MMP-2 Expression

ProMMP-2 significantly decreased when SK-MES-1 cells were exposed to hypoxic conditions at 6 h (normoxia 8032.6 ± 228.5 D.U. and hypoxia 2790.1 ± 186.8 D.U.; * *p* = 0.001). In contrast, proMMP-2 expression increased only slightly under hypoxia at 12 h compared with normoxia (3271.5 ± 187.9 and 2268.5 ± 267.6 D.U., respectively; ** *p* = 0.01) (Figure 1B). In addition, active MMP-2 levels decreased under hypoxia compared with normoxia at 6 h (25,258.1 ± 1399.5 and 35,575.2 ± 752.2 D.U., respectively; ^†^ *p* = 0.001) and 12 h (22,902.7 ± 359.6 and 26,371.8 ± 885.1 D.U., respectively; ^‡^ *p* = 0.002).

#### 2.1.4. CALU-1 Cell Line MMP-2 Expression

ProMMP-2 expression was significantly augmented under hypoxia compared with normoxia at 6 h (66,935.5 ± 4043.9 and 57,982.3 ± 2343.9 D.U., respectively; * *p* = 0.04) (Figure 1B). In contrast, proMMP-2 levels were lower when cells were cultured under hypoxic compared with normoxic conditions at 12 h (58,044.5 ± 3712.6 and 83,485.9 ± 2263.4 D.U., respectively; ** *p* = 0.001). No significant differences in active MMP-2 protein expression were observed between normoxic and hypoxic conditions at 6 and 12 h.

#### 2.1.5. SKLU-1 Cell Line MMP-2 Expression

SKLU-1 cells also expressed proMMP-2 under normoxic and hypoxic conditions at 6 and 12 h, but there were no significant differences between the two conditions (Figure 1B). In contrast, active MMP-2 levels were increased in cells cultured under hypoxia compared with normoxia at 6 h (49,137.4 ± 3632.2 and 32,492.1 ± 917.9 D.U., respectively; ^†^ *p* = 0.001) and 12 h (46,038.9 ± 4851.8 and 34,380.8 ± 2116.2 D.U., respectively; ^‡^ *p* = 0.02).

#### 2.1.6. MMP-2 Expression Among NSCLC Cells

The Kruskal–Wallis test revealed statistically significant differences in proMMP-2 expression between groups in normoxic (H = 24.86, df = 4, *p* < 0.001) and hypoxic (H = 30.73, df = 4, *p* < 0.001) conditions at 6 h (Figure 1C). Significant differences (adjusted “*p*” values) between cell lines were seen in A549 vs. SKMES-1 cells (* *p* = 0.0035), SKMES-1 vs. CALU-1 cells (** *p* = 0.00001), and CALU-1 vs. SKLU-1 cells (*** *p* = 0.0438) under normoxic conditions at 6 h. Differences between A427 vs. SK-MES-1 cells (^†^ *p* = 0.0133), SKMES-1 vs. A549 cells (^††^ *p* = 0.0005), A549 vs. SKLU-1 cells (^†††^ *p* = 0.0405), CALU-1 vs. SKMES-1 cells (^‡^ *p* = 0.00001), and SKLU-1 vs. CALU-1 cells (^‡‡^ *p* = 0.0031), were detected under hypoxia at 6 h.

Additionally, significant differences were observed between groups in normoxic (H = 26.55, df = 4, *p* < 0.001) and hypoxic (H = 23.11, df = 4, *p* < 0.001) conditions at 12 h. Significant differences were observed between A549 vs. SKMES-1 cells (* *p* = 0.0002), SKMES-1 vs. CALU-1 cells (** *p* = 0.0002), and CALU-1 vs. SKLU-1 cells (*** *p* = 0.0427) under normoxia, and between A427 vs. SKMES-1 cells (^†^ *p* = 0.039), A549 vs. SKMES-1 cells (^††^ *p* = 0.0008), and CALU-1 vs. SKMES-1 cells (^†††^ *p* = 0.0013) under hypoxia at 12 h.

Likewise, the Kruskal–Wallis test detected significant differences in active MMP-2 levels in normoxic conditions (H = 16.28, df = 4, *p* = 0.003), and differences between groups were observed in A427 vs. SKMES-1 cells (* *p* = 0.0126) and CALU-1 vs. A427 cells (** *p* = 0.0035) at 6 h (Figure 1C). In addition, the Kruskal–Wallis test showed statistically significant differences between groups (H = 11.00, df = 4, *p* = 0.027), but the post hoc analysis using Dunn’s test with Bonferroni adjustment for multiple comparisons revealed no significant differences between any of the groups under hypoxia at 6 h.

Similarly, significant differences among groups were detected in active MMP-2 expression (H = 15.62, df = 4, *p* = 0.004), with differences between A427 vs. CALU-1 cells (* *p* = 0.0306) and SKLU-1 vs. A427 cells (** *p* = 0.0075) in normoxic conditions at 12 h. Moreover, active MMP-2 expression differed significantly among groups (H = 31.52, df = 4, *p* < 0.001) under hypoxia at 12 h. The differences were detected between A427 vs. A549 cells (^†^ *p* = 0.00001), SKMES-1 vs. A549 cells (^††^ *p* = 0.0001), and SKLU-1 vs. A549 cells (^†††^ *p* = 0.0326) at 12 h in hypoxic conditions.

#### 2.1.7. MMP-2 Protein Expression over Time

ProMMP-2 and active MMP-2 protein expression at 6 h was compared with values obtained at 12 h under normoxic and hypoxic conditions. ProMMP-2 levels increased significantly in CALU-1 cells (*p* = 0.014), whereas they decreased in SKMES-1 cells (*p* = 0.001) at 12 h under normoxia. There were no significant differences in proMMP-2 expression between 6 and 12 h under hypoxia in all cell lines. In addition, active MMP-2 was lower at 12 h than at 6 h in SMES-1 cells under normoxic conditions (*p* = 0.001) and in A427 cells under hypoxic conditions (*p* = 0.028). Active MMP-2 levels were augmented at 12 h in comparison to 6 h in A549 cells under hypoxic conditions (*p* = 0.0003) (Table S1).

### 2.2. MMP-9 Expression

MMP-9 immunoblotting revealed a 96 kDa band corresponding to proMMP-9 (latent form) in all cell lines, with varying intensities under normoxic and hypoxic conditions at 6 and 12 h (Figure 2A). Additionally, a band with a molecular weight of about 85 kDa, corresponding to the active MMP-9 form, was observed in almost all cell lines under both normoxia and hypoxia. This band was not detected in A427 cells under normoxia at 12 h, nor under hypoxia at 6 and 12 h. Additionally, a band of about 130 kDa was visualized in SKMES-1 and SKLU-1 cells, which may correspond to MMP-9 dimers. The densitometric results for each cell line under normoxic and hypoxic conditions at 6 and 12 h are displayed below in Figure 2B. Figure 2C compares MMP-9 expression among NSCLC cells.

#### 2.2.1. A427 Cell Line MMP-9 Expression

ProMMP-9 expression was significantly augmented under hypoxia compared with normoxic conditions at 6 h (60,139.5 ± 2446.7 and 27,594.4 ± 639.4 D.U., respectively; * *p* = 0.04), and 12 h (56,891.4 ± 3837.8 and 14,417.1 ± 911.1 D.U., respectively; ** *p* = 0.03) in A427 cells (Figure 2B). In addition, the active MMP-9 form was observed only under normoxia at 6 h.

#### 2.2.2. A549 Cell Line MMP-9 Expression

ProMMP-9 expression was significantly increased in hypoxic compared with normoxic conditions at 12 h (49,104.3 ± 5525.5 and 4758.9 ± 674.4 D.U., respectively; * *p* = 0.01) in A549 cells (Figure 2B). No significant differences were observed at 6 h. Moreover, active MMP-9 levels augmented under hypoxia compared with normoxia at 6 h (16,910.4 ± 2777.1 and 9123.1 ± 1354.1 D.U., respectively; ^†^ *p* = 0.02), and at 12 h (71,570.5 ± 7661.3 and 11,468.1 ± 2445.6 D.U., respectively; ^‡^ *p* = 0.01).

#### 2.2.3. SKMES-1 MMP-9 Expression

SKMES-1 cells showed a significant decrease in proMMP-9 expression under hypoxic versus normoxic conditions at 6 h (7063.2 ± 316.6 and 9974.8 ± 622.8 D.U., respectively; * *p* = 0.002) (Figure 2B). No significant differences were observed between normoxic and hypoxic conditions at 12 h. Active MMP-9 protein expression was augmented under hypoxia compared with normoxia at 12 h (13,025.1 ± 912.3 and 8480.9 ± 784.1 D.U., respectively; ^†^ *p* = 0.005). No significant differences were observed between normoxia and hypoxia at 6 h.

#### 2.2.4. CALU-1 MMP-9 Expression

ProMMP-9 expression was diminished under hypoxia compared with normoxic conditions at 6 h (24,085.8 ± 952.1 and 27,088.1 ± 665.8 D.U., respectively; * *p* = 0.03) in CALU-1 cells (Figure 2B). In contrast, proMMP-9 expression increased under hypoxia compared with normoxia at 12 h (51,490.4 ± 1865.6 and 47,139.8 ± 1420.8 D.U., respectively; ** *p* = 0.03). Additionally, active MMP-9 expression did not differ significantly between normoxic and hypoxic conditions at 6 and 12 h.

#### 2.2.5. SKLU-1 MMP-9 Expression

ProMMP-9 expression did not differ significantly between normoxic and hypoxic conditions at 6 h in SK-LU-1 cells (Figure 2B). However, proMMP-9 expression decreased in hypoxic compared with normoxic conditions at 12 h (4703.7 ± 399.4 and 6601.2 ± 687.3 D.U., respectively; * *p* = 0.02). Active MMP-9 was augmented under hypoxia compared with normoxia at 6 h (44,455.1 ± 1250.9 and 35,790.3 ± 1149.1 D.U., respectively; ^†^ *p* = 0.001), but no significant differences were observed at 12 h.

#### 2.2.6. MMP-9 Expression Among NSCLC Cells

The Kruskal–Wallis test showed statistically significant differences in proMMP-9 expression between cell lines (H = 23.33, df = 4, *p* < 0.001) in normoxic conditions at 6 h (Figure 2C). Differences (adjusted “*p*” values) were detected among A427 vs. A549 cells (* *p* = 0.0055), A549 vs. CALU-1 cells (** *p* = 0.0013), and SKMES-1 vs. CALU-1 cells (*** *p* = 0.0075). In addition, significant differences in proMMP-9 expression between the cell groups (H = 18.34, df = 4, *p* = 0.001) were observed under hypoxic conditions at 6 h. Differences were seen between A427 vs. A549 cells (^†^ *p* = 0.0083), SKMES-1 vs. A427 cells (^††^ *p* = 0.03), and CALU-1 vs. A549 cells (^†††^ *p* = 0.0273).

Additionally, proMMP-9 levels differed significantly between cell lines (H = 20.02, df = 4, *p* < 0.001) under normoxic conditions at 12 h. Differences between groups were shown in A427 vs. A549 cells (* *p* = 0.0451), A549 vs. CALU-1 cells (** *p* = 0.0065), CALU-1 vs. SKMES-1 cells (*** *p* = 0.0069), and SKLU-1 vs. CALU-1 cells (^#^ *p* = 0.0411). Additionally, proMMP-9 expression differed significantly among cell lines under hypoxia at 12 h (H = 21.80, df = 4, *p* < 0.001). The differences were identified between A427 vs. SKLU-1 cells (^†^ *p* = 0.0092), A549 vs. SKLU-1 cells (^††^ *p* = 0.0229), and CALU-1 vs. SKLU-1 cells (^†††^ *p* = 0.0171).

Active MMP-9 levels differed significantly among cell lines (H = 46.80, df = 4, *p* < 0.001) under normoxic conditions at 6 h (Figure 2C). The differences were seen among A549 vs. CALU-1 cells (* *p* = 0.0047), A549 vs. SKLU-1 cells (** *p* = 0.0266), SKMES-1 vs. CALU-1 cells (*** *p* = 0.0001), and SKMES-1 vs. SKLU-1 (^#^ *p* = 0.0001). Significant differences in active MMP-9 expression under hypoxic conditions were also detected (H = 41.77, df = 3, *p* < 0.001) at 6 h, and differences were observed between SKMES-1 vs. CALU-1 cells (^†^ *p* = 0.0001), and SKMES-1 vs. SKLU-1 cells (^††^ *p* = 0.0001).

Likewise, significant differences in active MMP-9 levels between cell groups were seen (H = 40.53, df = 3, *p* < 0.001) in normoxic conditions at 12 h, with differences detected between A549 vs. CALU-1 cells (* *p* = 0.0119), SKMES-1 vs. CALU-1 cells (** *p* = 0.0001), and SK-LU-1 vs. SKMES-1 cells (*** *p* = 0.0001). Furthermore, active MMP-9 expression differed significantly under hypoxia at 12 h (H = 40.89, df = 3, *p* < 0.001). Differences between cell lines were reported between A549 vs. SKMES-1 cells (^†^ *p* = 0.0001), CALU-1 vs. SKMES-1 cells (^††^ *p* = 0.0001), and SKLU-1 vs. SKMES-1 cells (^†††^ *p* = 0.0002).

#### 2.2.7. MMP-9 Protein Expression over Time

Comparing proMMP-9 protein expression over 6 and 12 h revealed a significant increase at 12 h in CALU-1 cells (*p* = 0.008), and a decrease in A427, SKMES-1, and SKLU-1 cells (*p* ≤ 0.026) under normoxia. ProMMP-9 levels were found to be augmented in A549 and CALU-1 cells (*p* ≤ 0.028) and reduced in SKLU-1 cells (*p* = 0.001) at 12 h under hypoxia. Furthermore, active MMP-9 protein expression was elevated in A549 and SKMES-1 cells (*p* ≤ 0.01) and decreased in SKLU-1 cells (*p* = 0.001) under hypoxia at 12 h in comparison to 6 h (Table S2).

### 2.3. MMP-2 and MMP-9 Enzymatic Activity

The zymography assay revealed the presence of lytic bands corresponding to MMP-2 and MMP-9 enzymatic activity (Figure 3A). These bands were analyzed using densitometry to compare the enzymatic activity detected under normoxic and hypoxic conditions (Figure 3B). Figure 3C compares MMPs’ enzymatic activity among different NSCLC cell lines.

#### 2.3.1. A427 MMP Enzymatic Activity

MMP-2 enzymatic activity was significantly decreased under hypoxia compared to normoxic conditions at 6 h (81,017.5 ± 1453.3 and 108,700.5 ± 611.1 D.U., respectively; * *p* = 0.001), and 12 h (87,398.6 ± 586.5 and 118,804.8 ± 855.9 D.U., respectively; ** *p* = 0.001) in A427 cells (Figure 3B). Similarly, MMP-9 activity was significantly reduced under hypoxia when compared to normoxia at 6 h (3813.2 ± 601.3 and 10,599.6 ± 655.3 D.U., respectively; ^†^ *p* = 0.001), and 12 h (12,815.7 ± 76.4 and 21,675.4 ± 863.7 D.U., respectively; ^‡^ *p* = 0.001).

#### 2.3.2. A549 MMP Enzymatic Activity

MMP-2 enzymatic activity increased in hypoxic compared with normoxic conditions at 6 h in A549 cells (129,138.4 ± 3820.6 and 108,150.2 ± 2786 D.U., respectively; * *p* = 0.004) (Figure 3B). No significant differences were identified between hypoxia and normoxia at 12 h. MMP-9 enzymatic activity was significantly higher under hypoxia than under normoxia at 6 h (69,290.4 ± 1036.6 and 61,517.5 ± 1080.4 D.U., respectively; ^†^ *p* = 0.001) and 12 h (73,967.8 ± 2132.8 and 28,150.6 ± 821.6 D.U., respectively; ^‡^ *p* = 0.001.

#### 2.3.3. SKMES-1 MMP Enzymatic Activity

There were no significant differences in the enzymatic activity of both MMPs between hypoxic and normoxic conditions at 6 h in SKMES-1 cells (Figure 3B). In contrast, MMP-2 enzymatic activity was significantly increased under hypoxia compared with normoxia (157,553.9 ± 3208.8 and 125,224.7 ± 6029.9 D.U., respectively; * *p* = 0.001), while MMP-9 enzymatic activity under hypoxia was lower than that observed in normoxic conditions (89,584.2 ± 1963.8 and 99,315.3 ± 2569.3 D.U., respectively; ^†^ *p* = 0.01) at 12 h.

#### 2.3.4. CALU-1 MMP Enzymatic Activity

MMP-2 enzymatic activity significantly decreased in CALU-1 cells cultured under hypoxia versus normoxic conditions at 6 h (24,952.15 ± 594.5 and 74,440.7 ± 1018.6 D.U., respectively; * *p* = 0.001) (Figure 3B). In contrast, enzymatic activity was higher in hypoxic compared with normoxic conditions at 12 h (142,058.5 ± 2274.5 and 126,230.3 ± 1419.1 D.U., respectively; ** *p* = 0.001). MMP-9 enzymatic activity was significantly decreased under hypoxia with respect to normoxia at 6 h (2991.6 ± 292.8 and 10,435.1 ± 368.1 D.U., respectively; ^†^ *p* = 0.001), while it increased under hypoxia compared with normoxia at 12 h (15,497.4 ± 300.4 and 8525.4 ± 970.1 D.U., respectively; ^‡^ *p* = 0.001).

#### 2.3.5. SKLU-1 MMP Enzymatic Activity

MMP-2 enzymatic activity was diminished in hypoxic compared with normoxic conditions at 6 h (4545.8 ± 253.4 and 21,597.9 ± 1466.7 D.U., respectively; * *p* = 0.001), and 12 h (6110.9 ± 270.5 and 11,810.6 ± 464.1 D.U., respectively; ** *p* = 0.001) in SKLU-1 cells’ media (Figure 3B). Similar results were observed for MMP-9 enzymatic activity, which decreased in hypoxic versus normoxic conditions at 6 h (19,553.6 ± 1196.1 and 23,988.5 ± 659.9 D.U., respectively; ^†^ *p* = 0.008) and 12 h (44,217.8 ± 976.1 and 55,360.4 ± 774.3 D.U., respectively; ^‡^ *p* = 0.001).

#### 2.3.6. MMP Enzymatic Activity Among NSCLC Cells

The Kruskal–Wallis test demonstrated statistically significant differences in MMP-2 enzymatic activity between cell lines (H = 26.83, df = 4, *p* < 0.001) under normoxic conditions at 6 h (Figure 3C). Significant differences (adjusted “*p*” values) were detected between A549 vs. CALU-1 cells (* *p* = 0.0117), SKLU-1 vs. A549 cells (** *p* = 0.0001), and SKMES-1 vs. SKLU-1 cells (*** *p* = 0.0012). Additionally, MMP-2 enzymatic activity differed significantly among cell groups (H = 27.87, df = 4, *p* < 0.001) under hypoxia at 6 h. Differences were identified between A549 vs. SKLU-1 cells (^†^ *p* = 0.004), SKMES-1 vs. CALU-1 cells (^††^ *p* = 0.004), and SKLU-1 vs. SKMES-1 cells (^†††^ *p* = 0.0001).

Moreover, MMP-2 enzymatic activity differed significantly between groups under normoxic (H = 24.01, df = 4, *p* < 0.001) and hypoxic conditions (H = 27.72, df = 4, *p* < 0.001) at 12 h. Significant differences in normoxic conditions were observed between A427 vs. SKLU-1 cells (* *p* = 0.048), A549 vs. CALU-1 cells (** *p* = 0.0352), SKMES-1 vs. SKLU-1 cells (*** *p* = 0.0021), and CALU-1 vs. SKLU-1 cells (^#^ *p* = 0.0004), and in hypoxic conditions between A549 vs. SKMES-1 cells (^†^ *p* = 0.0045), SKMES-1 vs. SKLU-1 cells (^††^ *p* = 0.0001), and CALU-1 vs. SKLU-1 cells (^†††^ *p* = 0.0035).

Likewise, MMP-9 enzymatic activity differed significantly among cancer cell lines under normoxia (H = 26.52, df = 4, *p* < 0.001) and hypoxia (H = 26.58, df = 4, *p* < 0.001) at 6 h (Figure 3C). Differences in normoxic conditions were detected among A427 vs. A549 cells (* *p* = 0.0433), SKMES-1 vs. A427 cells (** *p* = 0.0005), A549 vs. CALU-1 cells (*** *p* = 0.0229), and CALU-1 vs. SKMES-1 cells (^#^ *p* = 0.0002), while significant differences were found between A427 vs. SKMES-1 cells (^†^ *p* = 0.0007), A549 vs. CALU-1 cells (^††^ *p* = 0.0184), and SKMES-1 vs. CALU-1 cells (^†††^ *p* = 0.0002) under hypoxia at 6 h.

In addition, MMP-9 enzymatic activity differed significantly between groups under normoxic (H = 27.87, df = 4, *p* < 0.001) and hypoxic (H = 27.87, df = 4, *p* < 0.001) conditions at 12 h. Significant differences were detected between A427 vs. SKMES-1 cells (* *p* = 0.004), SKMES-1 vs. CALU-1 cells (** *p* = 0.0001), and CALU-1 vs. SKLU-1 cells (*** *p* = 0.004) in normoxic conditions, and A427 vs. A549 cells (^†^ *p* = 0.004), SKMES-1 vs. A427 cells (^††^ *p* = 0.0001), and CALU-1 vs. SKMES-1 cells (^†††^ *p* = 0.004) under hypoxia at 12 h.

#### 2.3.7. MMP-2 and MMP-9 Enzymatic Activity over Time

Additionally, a comparison of MMP-2 enzymatic activity at 6 and 12 h revealed an increase in A427 and CALU-1 cells (*p* ≤ 0.003), while it decreased in A549 and SKLU-1 cells (*p* ≤ 0.003) under normoxia at 12 h. Moreover, MMP-2 enzymatic activity was augmented in A427, SKMES-1, CALU-1, and SKLU-1 cells (*p* ≤ 0.007) at 12 h, and reduced in A549 cells (*p* = 0.003) at 12 h under hypoxia. Similarly, MMP-9 enzymatic activity was higher in A427 and SKLU-1 cells (*p* ≤ 0.001), while it was lower in A549 cells (*p* = 0.003) at 12 h compared to 6 h in normoxic conditions. Furthermore, MMP-9 enzymatic activity was increased in A427, CALU-1, and SKLU-1 cells (*p* ≤ 0.001) at 12 h in comparison with 6 h under hypoxia (Tables S1 and S2).

### 2.4. HIF-1α and HIF-2α Expression in Lung Cancer Cell Lines

The hypoxic TME activates multiple signaling pathways, including the HIF pathway. HIFs regulate the transcription of molecules involved in cancer progression. Here, HIF-1α and HIF-2α protein expression was determined in the nuclear extract from NSCLC cells. HIF-1α (130 kDa band) and HIF-2α (120 kDa band) protein expression was assessed under normoxic and hypoxic conditions in all cell lines at 6 h and 12 h using Western blot analysis (Figure 4A,B). Higher-molecular-weight bands were also observed. These bands may correspond to HIFs bound to fragments of other nuclear molecules generated during the extraction process. Band intensities were analyzed by densitometry, and results comparing hypoxia and normoxia for each cell line are shown in Figure 4C and described below. Figure 4D compares the effects of hypoxia on HIF expression across NSCLC cell lines.

#### 2.4.1. A427 HIF Expression

HIF-1α increased in hypoxic compared with normoxic conditions at 6 h (39,635.9 ± 1846.6 and 28,141.7 ± 2714.1 D.U., respectively; * *p* = 0.003) in A427 cells (Figure 4C). In contrast, HIF-1α expression was lower under hypoxia than under normoxia at 12 h (32,693.7 ± 2128.7 and 43,463.7 ± 2563.4 D.U., respectively; ** *p* = 0.006). In addition, HIF-2α expression did not differ significantly between hypoxic and normoxic conditions at 6 h and 12 h.

#### 2.4.2. A549 HIF Expression

HIF-1α expression was significantly higher in hypoxic compared with normoxic conditions at 6 h (10,858.5 ± 628.7 and 6972.1 ± 393.7 D.U., respectively, * *p* = 0.001), and 12 h (18,915.1 ± 314.2 and 16,680.4 ± 454.4 D.U., respectively; ** *p* = 0.004) in A549 cells (Figure 4C). In contrast, HIF-2α expression did not differ significantly between hypoxia and normoxia at 6 and 12 h.

#### 2.4.3. SKMES-1 HIF Expression

HIF-1α expression was lower in hypoxic compared with normoxic conditions at 6 h (25,705.4 ± 2040.2 and 36,755.1 ± 2038.6 D.U., respectively; * *p* = 0.003) and 12 h (18,895.1 ± 1748.1 and 30,426.5 ± 2279.6 D.U., respectively; ** *p* = 0.003) in SKMES-1 cells (Figure 4C). No significant differences in HIF-2α expression were observed between hypoxic and normoxic conditions at 6 h. However, HIF-2α expression increased under hypoxia with respect to normoxia at 12 h (49,345.1 ± 795.5 and 32,033.2 ± 3227 D.U., respectively; ^†^ *p* = 0.008).

#### 2.4.4. CALU-1 HIF Expression

HIF-1α expression was lower under hypoxia compared to normoxia at 6 h (7377.7 ± 173.3 and 11,893.4 ± 739.9 D.U., respectively, * *p* = 0.001) (Figure 4C). In contrast, HIF-1α increased slightly under hypoxia compared with normoxia at 12 h (7947.2 ± 556.6 and 6109.1 ± 485.7 D.U., respectively; ** *p* = 0.04). HIF-2α expression was higher under normoxia compared with hypoxia at 12 h (28,460.1 ± 1856.7 and 9784.7 ± 255.4 D.U., respectively; ^†^ *p* = 0.01). No significant differences in HIF-2α expression were observed between normoxia and hypoxia at 6 h.

#### 2.4.5. SKLU-1 HIF Expression

HIF-1α expression was diminished under hypoxia compared with normoxic conditions at 6 h (35,404.9 ± 460.9 and 39,602.4 ± 366.2 D.U., respectively; * *p* = 0.001), but when cells were cultured for 12 h, HIF-1α expression increased under hypoxia in comparison with normoxia (37,851.6 ± 260.5 and 32,121.2 ± 202.6 D.U., respectively; ** *p* = 0.001) in SKLU-1 cells (Figure 4C). HIF-2α expression decreased in hypoxic compared with normoxic conditions at 6 h (20,635.4 ± 313.3 and 25,664.3 ± 216.9 D.U., respectively; ^†^ *p* = 0.001), and 12 h (22,208.6 ± 228.2 and 27,034.7 ± 346.7 D.U., respectively; ^‡^ *p* = 0.001).

#### 2.4.6. HIF Protein Expression Among NSCLC Cells

The Kruskal–Wallis test showed statistically significant differences in HIF-1α expression between NSCLC cells in normoxic (H = 37.68, df = 4, *p* < 0.001) and hypoxic (H = 35.88, df = 4, *p* < 0.001) conditions at 6 h (Figure 4D). The significant differences (adjusted “*p*” values) were detected among A427 vs. A549 cells (* *p* = 0.0064), A549 vs. SKMES-1 cells (** *p* = 0.0001), SKLU-1 vs. A549 cells (*** *p* = 0.0001), and CALU-1 vs. SKMES-1 cells (^#^ *p* = 0.0006) in normoxic conditions, and in A427 vs. A549 cells (^†^ *p* = 0.0034), CALU-1 vs. A427 cells (^††^ *p* = 0.0001), and SKLU-1 vs. CALU-1 cells (^†††^ *p* = 0.0011) under hypoxia at 6 h.

Moreover, cell groups differed significantly in HIF-1α expression under normoxic (H = 35.95, df = 4, *p* < 0.001) and hypoxic (H = 34.37, df = 4, *p* < 0.001) conditions at 12 h. Differences were seen between A427 vs. A549 cells (* *p* = 0.0014), CALU-1 vs. A427 cells (** *p* = 0.0001), and SKMES-1 vs. CALU-1 cells (*** *p* = 0.0057) in normoxic conditions, and between A427 vs. CALU-1 cells (^†^ *p* = 0.0001), and CALU-1 vs. SKLU-1 cells (^††^ *p* = 0.0001) hypoxic conditions at 12 h.

Likewise, statistically significant differences in HIF-2α expression between cell lines was detected in normoxic (H = 18.78, df = 4, *p* < 0.001) and hypoxic (H = 17.77, df = 4, *p* = 0.001) conditions at 6 h and differences between groups were reported in A549 vs. SKMES-1 cells (* *p* = 0.0472), and SKMES-1 vs. CALU-1 cells (** *p* = 0.0003) at normoxic conditions, and in SKMES-1 vs. CALU-1 cells (^†^ *p* = 0.0021), and SKLU-1 vs. SKMES-1 cells (^††^ *p* = 0.015) under hypoxia at 6 h.

In addition, significant differences in HIF-2α levels between cancer cell lines were observed in normoxic (H = 14.61, df = 4, *p* = 0.006) and hypoxic (H = 25.32, df = 4, *p* < 0.001) conditions at 12 h. The differences detected were between A427 vs. A549 cells (* *p* = 0.0054), and A549 vs. SKMES-1 cells (** *p* = 0.0167) in normoxic conditions, and among A427 vs. A549 cells (^†^ *p* = 0.0169), CALU-1 vs. A427 cells (^††^ *p* = 0.0017), A549 vs. SKMES-1 cells (^†††^ *p* = 0.0098), and SKMES-1 vs. CALU-1 cells (^‡^ *p* = 0.0012) in hypoxic conditions at 12 h.

#### 2.4.7. HIF Protein Expression over Time

HIF-1α protein expression was augmented in A427 and A549 cells (*p* ≤ 0.001), while its expression decreased in SKMES-1, CALU-1, and SKLU-1 (*p* ≤ 0.03) at 12 h with respect to 6 h in normoxic conditions. In addition, HIF-1α expression increased in A549 and SKLU-1 cells (*p* ≤ 0.004) at 12 h, whereas its expression declined in A427 and SKMES-1 (*p* = 0.03) under hypoxia (Table S3).

Similarly, HIF-2α protein expression increased in CALU-1 and SKLU-1 cells (*p* ≤ 0.014) and diminished in A549 cells (*p* = 0.003) at 12 h under normoxic conditions. Furthermore, HIF-2α protein expression was enhanced in SKLU-1 cells (*p* = 0.004) and decreased in A549 and CALU-1 cells (*p* ≤ 0.014) at 12 h compared with 6 h in hypoxic conditions (Table S3).

### 2.5. MMP and HIF Protein Expression in NSCLC Cell Lines

There may be an association between HIFs’ expression and MMPs’ synthesis and enzymatic activity in cells exposed to hypoxia. For example, HIF-1α protein expression was augmented in A427, in which proMMP-2, active MMP-2, and proMMP-9 were also increased, whereas in A549 cells, the increase in HIF-1α was accompanied by an increase in actMMP-9, MMP-2 enzymatic activity, and MMP-9 enzymatic activity at 6 h in hypoxic conditions (Figure 5A). HIF-2α synthesis did not increase when cells were cultured for 6 h under hypoxia.

Likewise, HIF-1α was augmented in A549 cells, with an increase in active MMP-2, proMMP-9, active MMP-9, and MMP-9 enzymatic activity, while elevated levels of HIF-1α were also observed in CALU-1 cells together with an increase in proMMP-9 protein expression and MMP-2 and MMP-9 enzymatic activity at 12 h under hypoxia compared to normoxic conditions (Figure 5B). Interestingly, although HIF-1α decreased in A427 cells at 12 h, proMMP-2 and proMMP-9 expression remained elevated under hypoxic conditions compared with normoxic conditions.

Additionally, HIF-2α protein expression was elevated in SKMES-1 cells after 12 h of hypoxia, along with increased proMMP-2 and active MMP-9 protein expression and MMP-2 enzymatic activity.

## 3. Discussion

MMPs play an important role in cancer evolution, from the initial events of metastasis to the formation of a metastatic lesion in a distant organ; therefore, they have been associated with the metastatic potential of neoplastic cells [10].

In the present work, we examined the expression and enzymatic activity of MMP-2 and MMP-9 in the culture medium from five different NSCLC cell lines grown under normoxic or hypoxic conditions. MMP expression and enzymatic activity differed among the cell lines studied. Moreover, hypoxia did not induce parallel changes in MMP expression and enzymatic activity. For example, in A427 cells, proMMP-2 and active MMP-2 increased, but enzyme activity decreased under hypoxia at 6 h. Additionally, the expression of both forms of MMP-2 may have remained unaffected by hypoxic conditions while enzymatic activity augmented in A549 cells, or expression decreased while enzymatic activity remained unchanged between normoxic and hypoxic conditions in SKMES-1 cells at 6 h. Furthermore, increased active MMP-2 expression was not reflected in augmented enzymatic activity, as observed in A427 and SKLU-1 cells at 6 h and in A549 and SKLU-1 cells at 12 h under hypoxia. In contrast, enhanced expression of both forms of MMP-9 was accompanied by increased enzymatic activity in A549 cells at 12 h under hypoxia. In addition, SKLU-1 cells showed augmented active MMP-9 and diminished enzymatic activity at 6 h, and SKMES-1 cells showed increased active MMP-9 and decreased enzymatic activity at 12 h.

In this regard, there are other cofactors and molecules involved in MMP activation whose function may be affected by hypoxic conditions, such as low pH, characteristic of the hypoxic TME, reactive oxygen species (ROS) levels, plasmin, furin, other MMPs like MMP-3 and MMP-14, and the metalloproteinase inhibitors TIMP-1 and TIMP-2, which participate in the activation of proMMP-2 and proMMP-9 [7,11].

Experiments on MMP expression have been conducted using NSCLC cell lines, particularly A549 and H1299 (large cell carcinoma). For example, MMP-9 expression was upregulated in both cell lines when cells overexpressed phosphoinositide 3-kinase (PI3K) catalytic subunit gamma (PI3KCG), with higher MMP-9 expression in A549 than in H1299 cells [12]. In addition, the role of Guanine Nucleotide-Binding Protein-Like 3-Like Protein (GNL3L) in cancer metastasis was examined in both cell lines [13]. Inhibiting GNL3L expression decreased cell migration and invasion, with a greater effect in A549 cells. Moreover, GNL3N was involved in upregulating NF-κB, Slug, MMP-2, and MMP-9 synthesis in this cell line.

Studies on MMP expression in NSCLC cell lines other than A549 cells are scarce. For instance, Eurycomanone (ECN) inhibited MMP-2 protein expression more strongly in CALU-1 than in A549 cells, although MMP-2 synthesis induced by TGF-β1 was higher in A549 than in CALU-1 cells without ECN [14]. Similarly, differences in MMP-9 mRNA expression and protein levels were identified in five NSCLC cell lines, including SKMES-1, with the most significant differences observed in A549 cells [15].

Differences in MMP expression in our work and in other authors’ studies using NSCLC cell lines may reflect genetic or epigenetic variation, as occurs in primary tumors, where heterogeneity among cellular subclones leads to distinct responses to TME conditions and therapeutic approaches [16,17,18].

Likewise, several MMPs have been identified in lung cancer tissue and blood samples. For example, Iniesta P. et al. found increased MMP-2 and MMP-9 expression in lung cancer tissue samples compared with normal tissue, but no correlation with TNM stage [19]. Additionally, lymph node metastasis was associated with increased net MMP-2 enzymatic activity. Likewise, Guo C.B. et al. identified a relationship between advanced NSCLC stages and MMP-2 and MMP-9 expression in tissue and blood samples, and also considered their prognostic value [20]. Moreover, MMP-2 expression has been reported to be higher when CD44st is overexpressed, and its upregulation was associated with advanced stages of the disease, including lymph node invasion [21].

In addition, patients at advanced stages (IIIA–IV) showed high MMP-2 expression in tissue [22]. This increase was associated with poorer survival outcomes, while low MMP-2 expression was found in patients with better survival rates. Furthermore, MMP quantification after oncologic lung resection surgery revealed an association between low MMP-2 and MMP-3 blood levels, and low MMP-7 and MMP-9 bronchoalveolar lavage levels, with longer survival and recurrence-free time [23]. Moreover, data from GSE31210 and the Cancer Genome Atlas indicate that overexpression of MMP-1, MMP-3, MMP-9, MMP-11, MMP-12, and MMP-13 in stage I lung adenocarcinoma is associated with worse prognosis and shorter survival [24]. Interestingly, these datasets also showed increased HIF-1α signaling.

Despite these findings, the correlation between their expression and tumor node metastasis (TNM) stage, as well as their prognostic value, remains unclear. Therefore, analyzing MMPs across different NSCLC cell lines might support their clinical diagnostic value.

On the other hand, hypoxia is implicated in cancer progression by regulating the different steps that occur during the metastatic process, such as the epithelial–mesenchymal transition (EMT), migration, angiogenesis, immune surveillance evasion, tumor cells’ intravasation and extravasation, and formation of the premetastatic niche in a distant tissue [8,25]. Because MMPs are involved in the initial events of the metastatic cascade, the hypoxic TME can induce their expression.

Here, different NSCLC cell lines were exposed to a hypoxic environment for 6 and 12 h, and the expression of HIF-1α and HIF-2α was analyzed. Both HIFs were expressed under normoxic conditions in all cell lines. In this context, HIF-1α can be stabilized even under normal oxygen levels, thereby regulating its target genes as an adaptive response in neoplastic cells [26]. Therefore, it is not surprising to find HIFs in tumor cells when grown under normal oxygen conditions. Additionally, HIF-1α expression was higher in NSCLC tissue samples than in normal tissue, particularly near necrotic areas in squamous cell carcinomas [27,28,29,30,31]. Interestingly, HIF-1α has also been identified in non-necrotic zones from adenocarcinoma tissue samples [27].

HIF-1α expression was increased in some cells under hypoxia at 6 h (A427 and A549 cells) or at 12 h (A549, SKILU-1, and CALU-1). If hypoxic exposure time were increased, HIF-1α protein expression in the other cell lines would also increase [32]. In addition, HIF-1α protein expression in A427 cells was augmented under hypoxia at 6 h but not at 12 h. Similarly, Uchida et al. reported that HIF-1α protein levels were higher at 4 h, followed by a decrease to basal concentrations at 12 h in A549 cells [33]. This effect of prolonged hypoxia exposure can be attributed to negative regulation of HIF-1α protein expression by increased natural antisense HIF-1α (aHIF), leading to transcriptional destabilization of HIF-1α [33]. In the present work, this response to longer hypoxic exposure periods was observed in A427 and SKMES-1 cells, but not in A549 cells, in which HIF-1α protein expression increased at 12 h under hypoxic conditions.

Likewise, HIF-2α expression has been identified in NSCLC tissue samples [34,35]. In the current study, HIF-2α expression did not increase in any cell line after 6 h of hypoxia exposure. However, it was enhanced in SKMES-1 cells at 12 h. Unlike the effects of hypoxia on HIF-1α expression at 12 h, HIF-2α appears to require longer periods of hypoxia to be expressed in response to moderate or chronic low-oxygen concentrations [33,34]. In contrast, HIF-1α expression increased after 4 h in response to acute and severe hypoxia during the first hours of hypoxic conditions in the TME.

HIF-1α upregulation is accompanied by increased VEGF and MMP-9 expression, both associated with angiogenesis [28,29,30]. Moreover, experiments in A549 cells under hypoxia for 4, 24, and 48 h showed increased MMP-2 and MMP-9 levels, associated with HIF-1α upregulation [36,37,38]. Here, a correlation between HIF-1α expression and MMP-2 and MMP-9 expression and enzymatic activity was identified mainly in A427 and A549 cells at 6 h and in A549, SKLU-1, and CALU-1 cells at 12 h. However, current findings represent co-expression observations; therefore, it cannot be stated that either HIF-1α or HIF-2α directly regulates MMP-2 and MMP-9 expression. To establish a direct relationship between HIF and MMP expression, further research is required; for instance, the use of siRNA or specific HIF inhibitors.

Likewise, to our knowledge, an HRE site has not been identified in the MMP-2 or MMP-9 promoter regions. Therefore, the effects of hypoxia on MMP-2 and MMP-9 protein expression and enzymatic activity observed in the present work cannot be attributed to direct HIF control. For example, hypoxia might induce MMP transcription in a HIF-independent manner through transcription factors such as NF-κB, STAT, sHH-Gli1, AP-1, and Est-1 [39]. Moreover, hypoxia activates the HIF-1α/IL-6/JAK/STAT/MMPs axis that promotes the expression of MMP-1, MMP-2, MMP-3, and MMP-9 [40]. In this context, HIF and MMP expression have been associated with therapy response in NSCLC patients. For example, the tyrosine kinase inhibitor (TKI), gefitinib, upregulates IL-6 under hypoxic conditions, activating the IL-6/JAK/STAT/MMPs axis and inducing gefitinib resistance and cancer progression [41]. Similarly, resistance to the ALK inhibitor crizotinib can be due to HIF-1α and Slug overexpression under hypoxic conditions [42].

Likewise, tissue samples from colorectal cancer (CRC) patients showed overexpression of MMP-2, HIF-1α, and zinc finger protein 384 (ZFP384), correlating with poor survival outcomes. The molecular mechanism involves hypoxia-induced activation of HIF-1α, which in turn induces ZFP384 transcription; ZFP384 then binds to the MMP-2 promoter site [43].

Additionally, HIFs may regulate the expression of other molecules involved in MMP synthesis and enzymatic activity. For instance, MMP-14, which is implicated in proMMP-2 activation, has a binding site for HIF-2α at its proximal promoter [44].

In summary, the differences detected in MMP and HIF expression in response to hypoxia in the present study must depend on genetic characteristics acquired by each cell during the process of carcinogenesis, other than the KRAS mutations shared by almost all the cell lines studied, with the exception of SKMES-1 cells, or TP53 present in SKMES-1 and CALU-1 cells. Furthermore, the cell lines were derived from primary tumors, where they had to adapt to the characteristics of their own TME. Therefore, the genetic and epigenetic changes thus acquired may influence their response to the hypoxic conditions used in the present study.

## 4. Materials and Methods

### 4.1. Cell Culture

Human NSCLC cell lines were acquired from the American Type Culture Collection (ATCC) (Manassas, VA, USA): CALU-1 (ATCC-HTB-54) and SKMES-1 (SK-MES-1, ATCC-HTB-58) squamous cell carcinomas; A549 (ATCC-CCL-185), A427 (ATCC-HTB-53), and SKLU-1 (SK-LU-1, ATCC-HTB-57) adenocarcinomas. The A549 cell line was cultured in Dulbecco’s Modified Eagle Medium (DMEM) (Gibco, Waltham, MA, USA), while the other cell lines were cultured in Roswell Park Memorial Institute (RPMI)-1640 medium (Gibco, Waltham, MA, USA). Both media were supplemented with 100 U/mL penicillin, 100 μg/mL streptomycin, 2.5 mg/mL amphotericin B, and 10% fetal bovine serum (FBS) (Gibco, Waltham, MA, USA). Cells were cultured in a humidified atmosphere containing 5% CO_2_ and 95% air at 37 °C. No Mycoplasma contamination was detected in any cell culture.

### 4.2. Hypoxia Assays

Cells (0.6 × 10^6^ cells/mL) were placed in 75 cm^2^ culture flasks, and when they reached 80% confluence, the medium was replaced with medium without FBS, and hypoxia or normoxia experiments were performed: 6 flasks for normoxia at 6 h, 6 flasks for hypoxia at 6 h, 6 flasks for normoxia at 12 h, and 6 flasks for hypoxia at 12 h. A total of 24 flasks per experiment for each cell line were tested. Three experiments were carried out for each condition. For hypoxia experiments, cells were cultured in a sealed modular incubator chamber (Billups-Rothenberg Inc., San Diego, CA, USA) containing 1% O_2_, 5% CO_2_, and 94% N_2_ in a humidified atmosphere at 37 °C for 6 and 12 h. Cells cultured in medium without FBS in a humidified atmosphere containing 5% CO_2_ and 95% air at 37 °C for 6 and 12 h were taken as controls (normoxic conditions). Cell viability was not affected after growing cells under hypoxia for 6 and 12 h. Therefore, the observed changes in MMP and HIF expression are likely due to hypoxia rather than cell death.

At the end of incubation, the medium and cells from six cultured flasks, one from each condition, were collected and pooled. The medium was concentrated by lyophilization in a freeze-dryer system (Labconco Lyph-Lock 4.5L, Labconco, Cor, Kansas City, MO, USA). Medium protein concentration was determined by the bicinchoninic acid protein method (Pierce Chemical Co., Rockford, IL, USA) [45].

The medium from each experiment was used for immunoblotting and zymography assays.

The cells were used to extract nuclear proteins using a nuclear extraction kit according to the manufacturer’s instructions (Abcam, Cambridge, MA, USA). Protein concentration in the nuclear extract was measured using the bicinchoninic acid protein assay.

### 4.3. Immunoblotting Assay

Electrophoresis was performed on 8% sodium dodecyl sulfate (SDS)-polyacrylamide gels (PAGE) under reducing conditions with 5% 2-mercaptoethanol. An equal amount of protein (30 µg) from the nuclear extract or medium samples was added per lane, and the gels were incubated at 100 °C for 10 min. Proteins were then transferred to polyvinylidene difluoride (PVDF) (Bio-Rad Laboratories, INC, Hercules, CA, USA) membranes, blocked with 2.5% non-fat dry milk in 100 mM Tris-HCl buffer, pH 7.5, containing 150 mM NaCl and 0.1% Tween-20 (TTBS buffer), and incubated with the corresponding primary antibody for 90 min at room temperature. Primary antibody concentrations were 7 µg/mL anti-MMP-9 mouse monoclonal antibody (BioLegend, San Diego, CA, USA); 10 µg/mL anti-MMP-2 mouse monoclonal antibody; 4 µg/mL rabbit monoclonal anti-HIF-1α; and 4 µg/mL mouse monoclonal anti-HIF-2α antibody. Four µg/mL anti-histone-H3 was used as a loading control for HIF Western blots (Abcam, Cambridge, MA, USA). Total protein quantification was used to normalize MMP Western blots.

Unbound antibodies were eliminated by washing the membranes with TTBS buffer. Membranes were then treated with the VECTASTAIN ABC kit (Vector Laboratories, Burlingame, CA, USA) according to the kit instructions. Bands visualized in the blotting assay were analyzed by densitometry using Kodak Digital Science ID Image Analysis Software, version 2.0 (Kodak, Rochester, NY, USA), which measures band area and intensity. The results were expressed as densitometry units (D.U.). Two Western blots were performed for each protein analyzed in each experiment; each experiment was carried out three times, resulting in an “n” of six Western blots.

To compare different Western blot results from each cell line, normalization of total protein amount quantification was carried out.

### 4.4. Zymography Assay

MMP-2 and MMP-9 enzymatic activity was examined using 8% SDS-PAGE containing 0.01% gelatin from porcine skin (Sigma-Aldrich Co., St. Louis, MO, USA). Total protein amount quantification was used for normalization in the zymography assay. An equal amount of protein (30 µg) from medium samples under non-reducing conditions was added per lane. HT-1080 (ATCC-CCL-121) fibrosarcoma cell medium was used as an enzymatic activity control. After electrophoresis, gels were washed twice with 2.5% Triton X-100 for 30 min each, and then incubated in Tris-HCl buffer containing 50 mmol/L Tris-HCl, 5 mmol/L ZnCl_2_, 0.15 mmol/L NaCl, and 20 mmol/L CaCl_2_, pH 7.4, for 24 h. After incubation, gels were stained with 0.05% Coomassie blue G-250 (Bio-Rad Laboratories, Inc., Hercules, CA, USA). The enzymatic (gelatinase) activity was visualized as transparent bands against a blue background. Bands were analyzed by densitometry. Two zymograms were performed in each experiment; each experiment was carried out three times, resulting in an “n” of six zymograms. To compare different zymogram results from each cell line, normalization of total protein amount quantification was carried out.

### 4.5. Statistical Analysis

SPSS version 20.0 (IBM Corp., Armonk, NY, USA) was used for statistical analysis. Non-parametric statistical analysis was employed since the data did not follow a normal distribution. The Mann–Whitney U test for pairwise comparisons was used to analyze densitometry results. Six separate Western blots or zymograms obtained from three independent experiments performed under normoxic and hypoxic conditions for each cell line were analyzed, and values were expressed as mean ± standard error (SE) of the mean from n = 6. Statistical significance was considered as *p* < 0.05. Comparisons between cell lines were examined using the Kruskal–Wallis ANOVA by ranks test with a multiple comparison of mean ranks followed by Dunn’s post hoc test with Bonferroni adjustment for multiple comparisons. Statistical significance (adjusted *p*-values) was considered as *p* < 0.05 for differences among cell lines.

## 5. Conclusions

Our preliminary findings reveal differences in MMP-2, MMP-9, HIF-1α, and HIF-2α protein expression between NSCLC cell lines under both normoxic and hypoxic conditions. In addition, changes in MMP expression were not similar to changes in their enzymatic activity. Therefore, those designing new therapies to halt metastasis must consider not only increased MMP expression but also their function, as reflected in increased enzymatic activity. Therefore, therapies designed to interfere with the metastatic process must consider not only MMP expression but also their enzymatic activity, as well as the effects of hypoxia through HIF-dependent and HIF-independent pathways in the TME.

## 6. Limitations and Future Perspectives

In this work, the co-expression of two MMPs and HIFs was identified. However, to confirm the role of HIFs in MMP-2 and MMP-9 expression under the hypoxic conditions studied, HIF-specific inhibitors or siRNAs are required. Furthermore, to better understand the role of hypoxia in MMP expression and enzymatic activity in NSCLC progression, it is necessary to conduct assays with longer hypoxia exposures and examine its effects not only on MMP-2 and MMP-9, but also on molecules involved in their expression and enzymatic activity regulation.

## Figures and Tables

**Figure 1:**
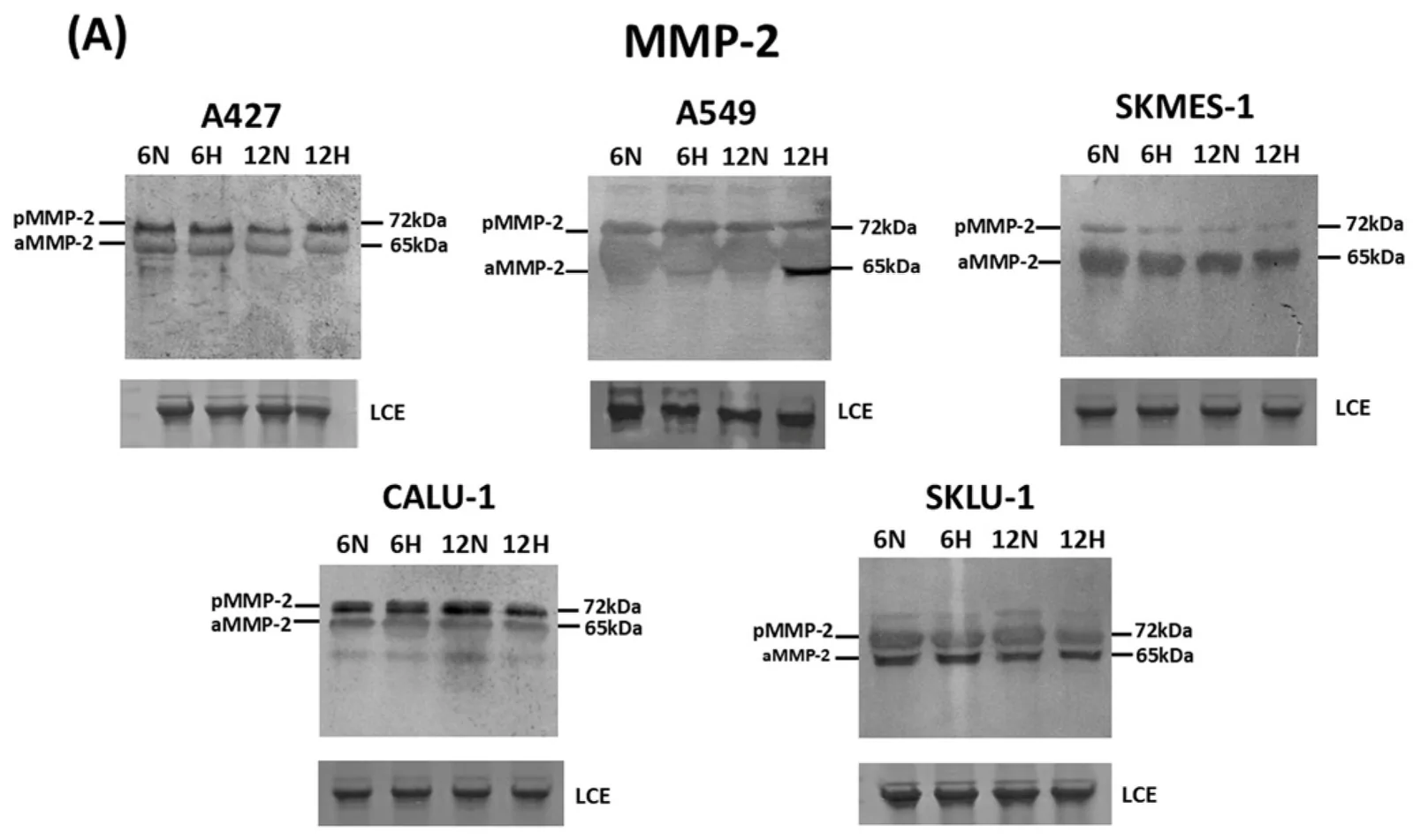

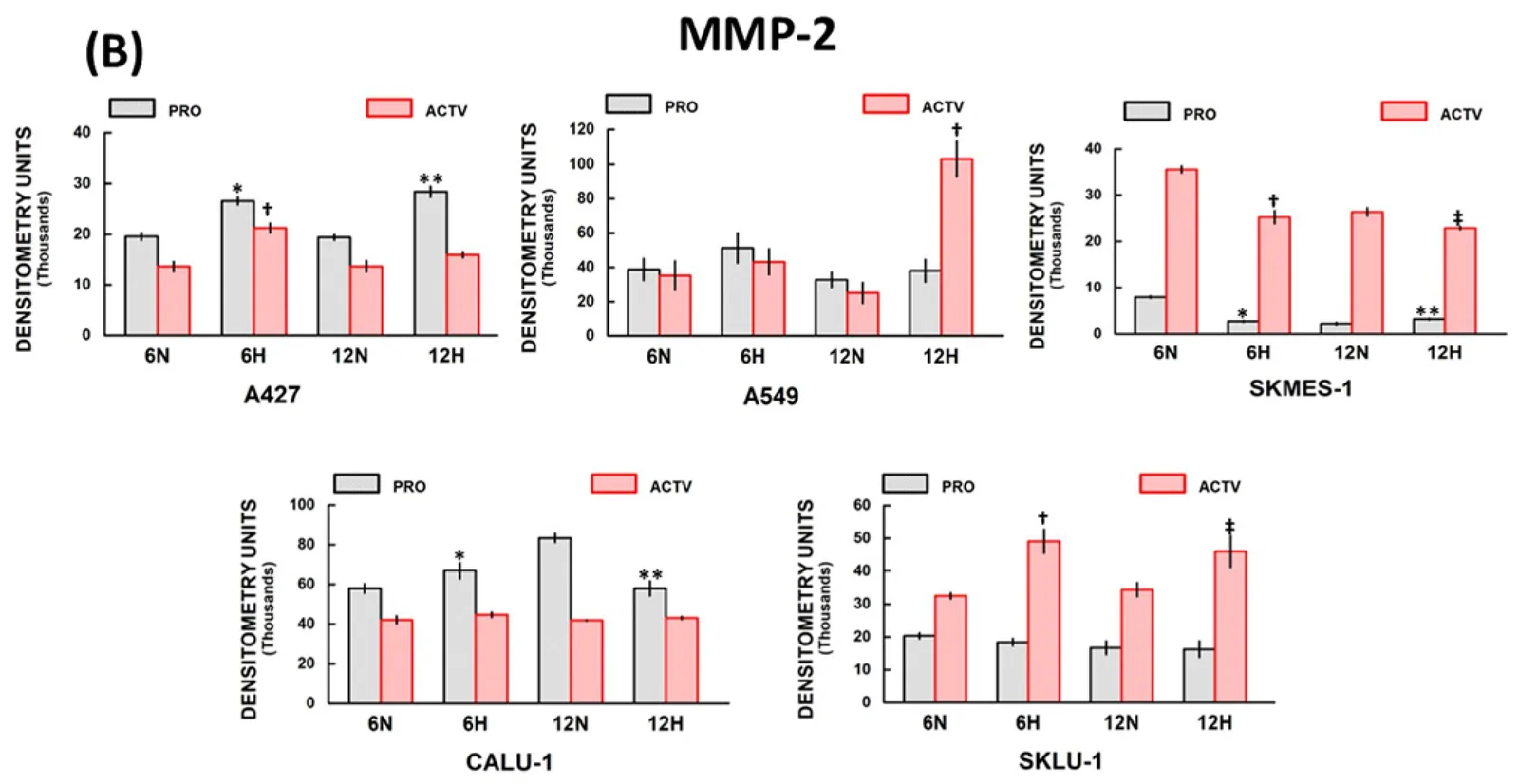

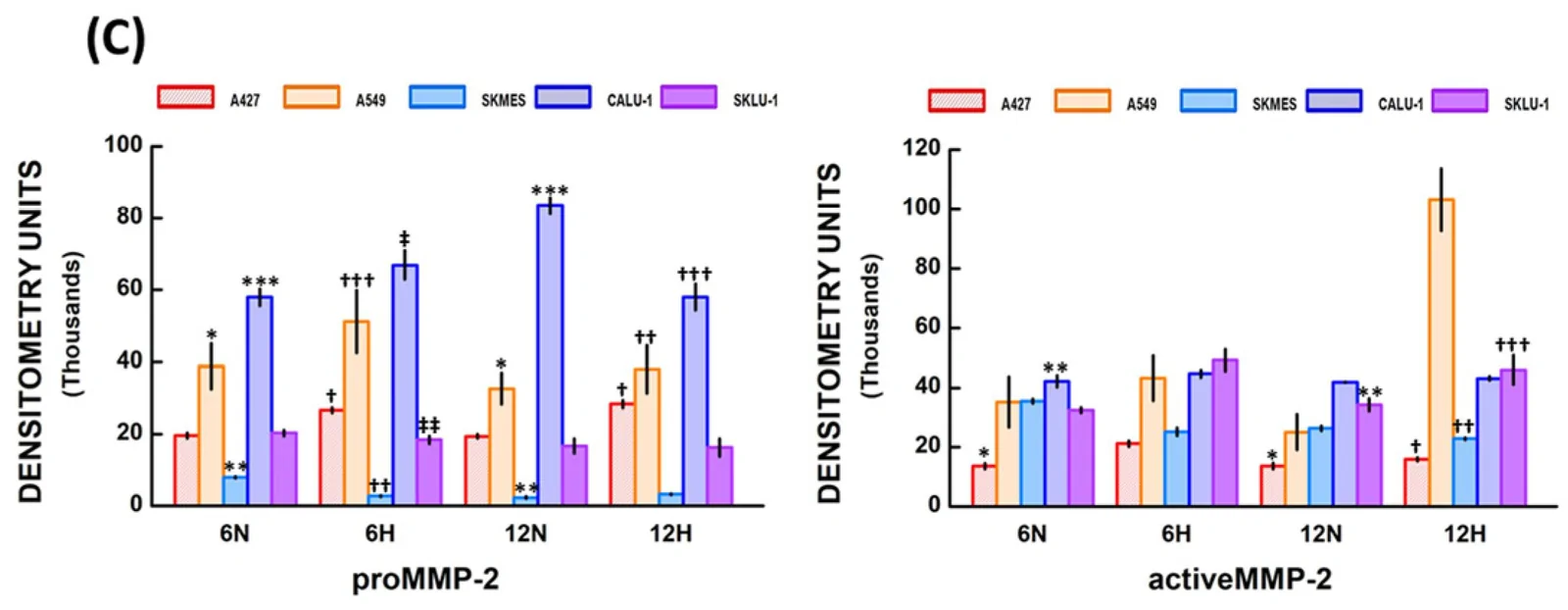
MMP-2 expression in different lung cancer cell lines grown under normoxic and hypoxic conditions for 6 and 12 h: (**A**) MMP-2 latent (pMMP-2) and active (aMMP-2) forms were expressed in all lung cancer cells; (**B**) Densitometry analysis showed significant differences in MMP-2 expression between cells grown in hypoxic (H) and normoxic (N) conditions at 6 and 12 h. In some cells, MMP-2 expression increased, whereas in others it decreased under hypoxia; (**C**) There were significant differences in proMMP-2 and active MMP-2 protein expression between NSCLC cell lines under normoxic (N) and hypoxic (H) conditions at 6 and 12 h. Bars represent mean ± standard error of the mean of 6 Western blots. Significant differences between cell lines are indicated by * *p*, ** *p*, *** *p*, ^†^ *p*, ^††^ *p*, ^†††^ *p*, ^‡^ *p*, ^‡‡^ *p*; for further explanation refer to Section 2.1.6. “MMP-2 expression among NSCLC cells”.Total protein quantification was used to normalize MMPs’ Western blots. Abbreviations: LCE = loading control electrophoresis (30 µg of protein per lane), MMP = matrix metalloproteinase, NSCLC = non-small cell lung cancer.

**Figure 2:**
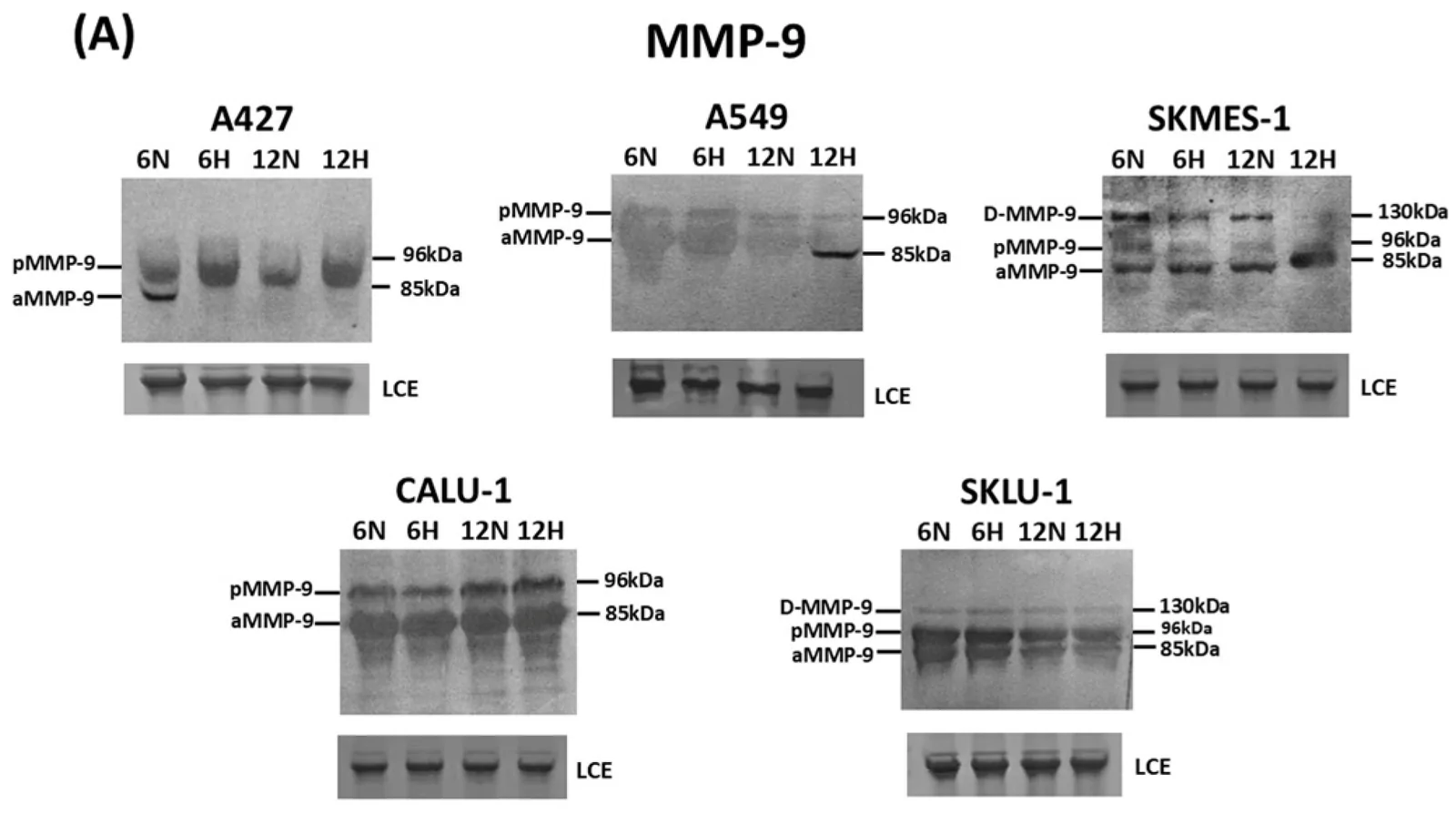

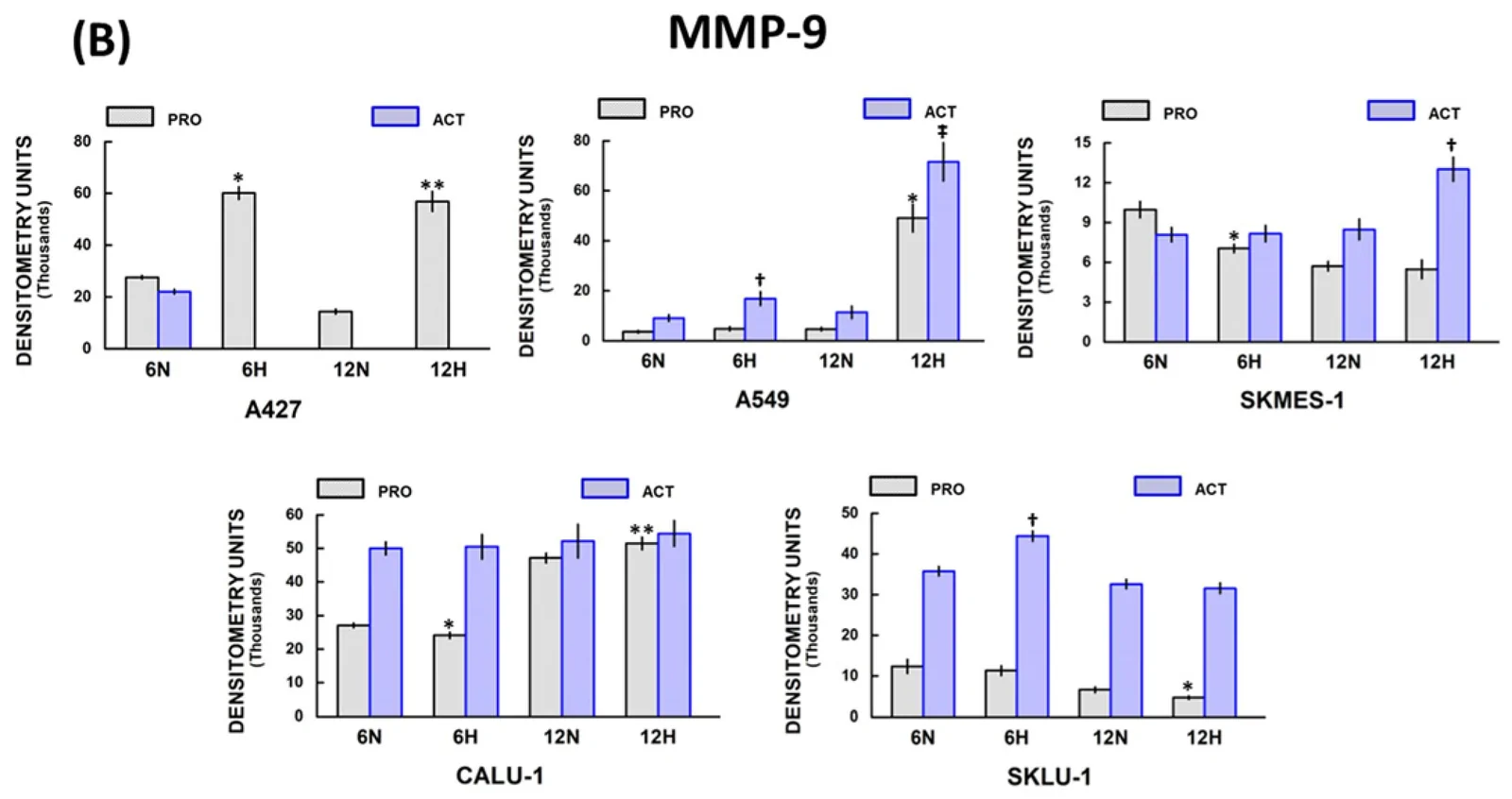

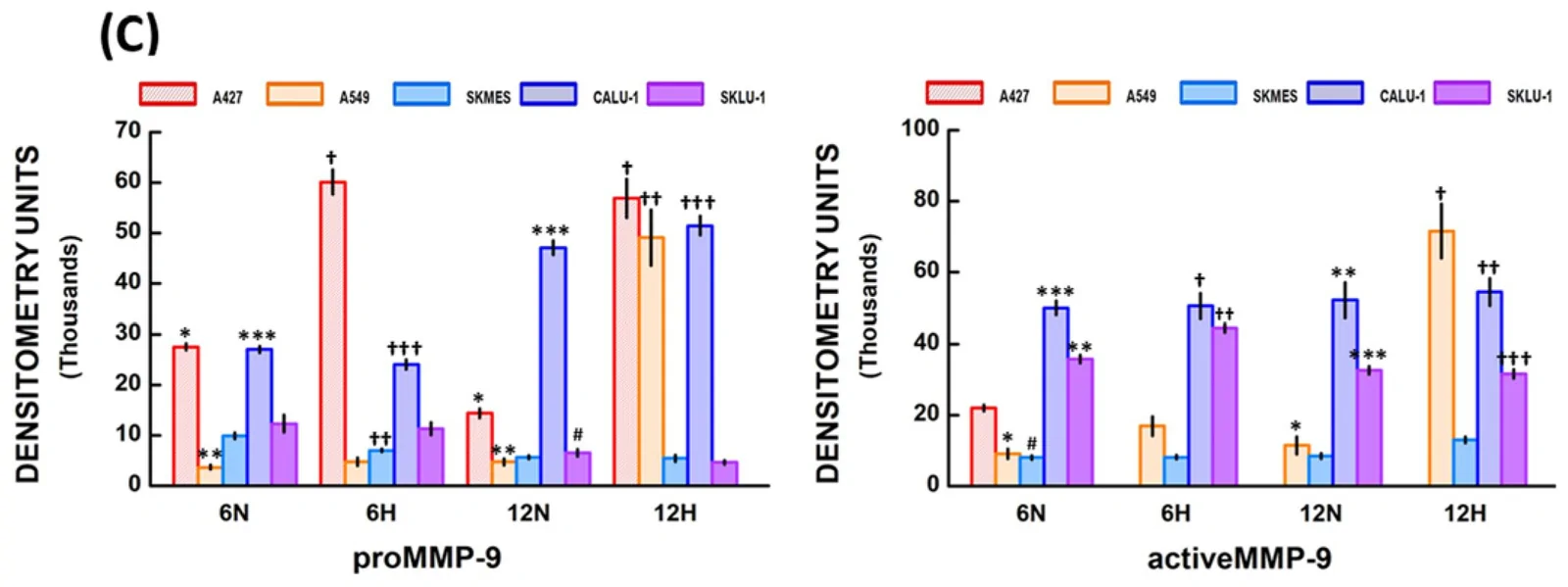
MMP-9 expression in lung cancer cells grown in hypoxic conditions: (**A**) Bands corresponding to proMMP-9 and active MMP-9 were detected in almost all cell lines. MMP-9 dimers (D-MMP-9) were also observed in SKMES-1 and SKLU-1 cells; (**B**) The densitometry analysis showed differences in proMMP-9 and active MMP-9 between normoxic (N) and hypoxic (H) conditions in all cell lines at 6 and 12 h; (**C**) Significant differences in proMMP-9 and active MMP-9 expression among NSCLC cell lines were observed in normoxic (N) and hypoxic (H) conditions at 6 and 12 h. Bars represent mean ± standard error of the mean of 6 Western blots. Significant differences between cell lines are indicated by * *p*, ** *p*, *** *p*, ^†^ *p*, ^††^ *p*, ^†††^ *p*, ^‡^ *p*, ^#^ *p*; for further explanation refer to Section 2.2.6. “MMP-9 expression among NSCLC cells”.Total protein quantification was used to normalize MMPs’ Western blots. Abbreviations: LCE = loading control electrophoresis (30 µg of protein per lane), MMP = matrix metalloproteinase, NSCLC = non-small cell lung cancer.

**Figure 3:**
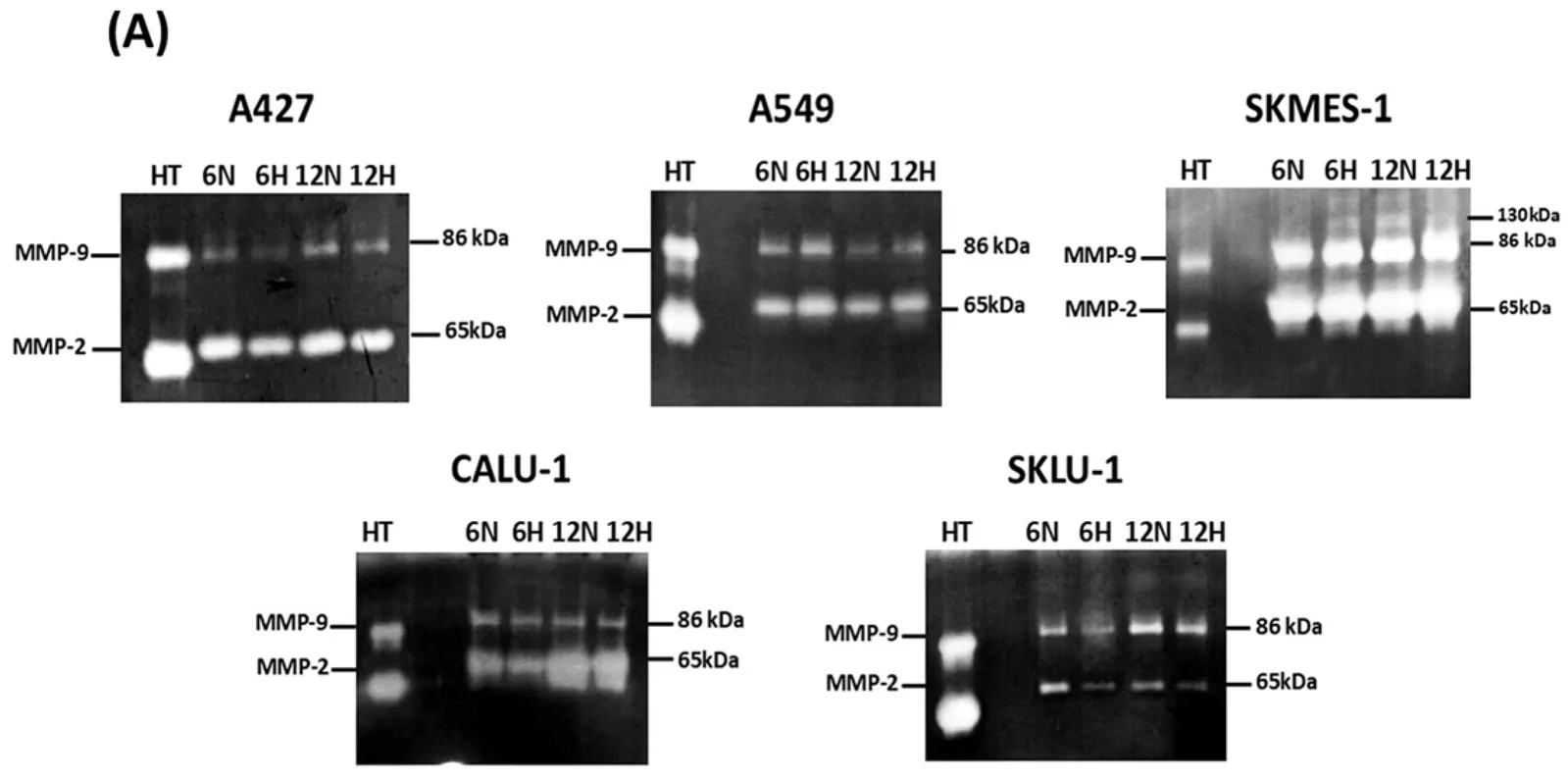

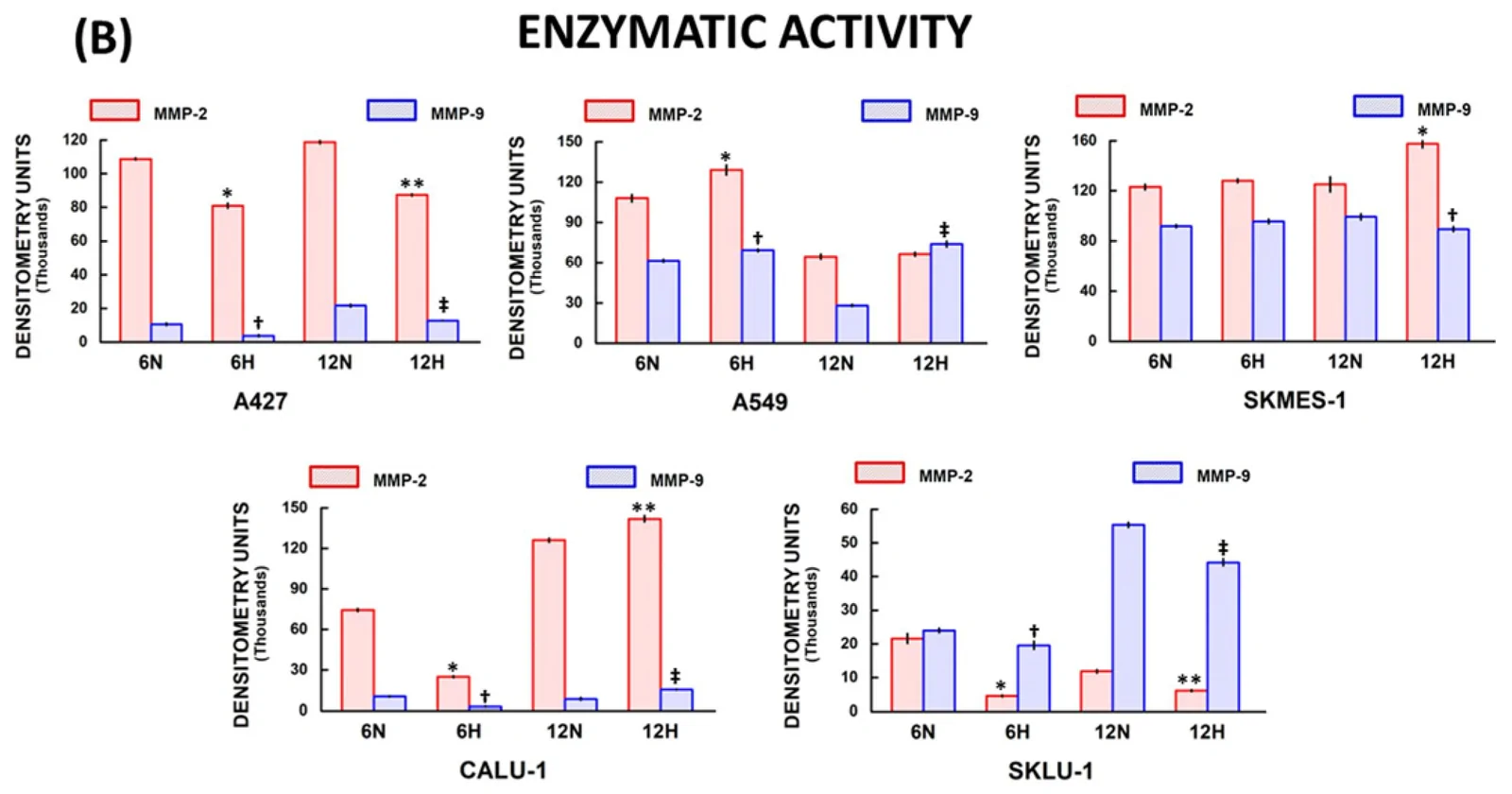

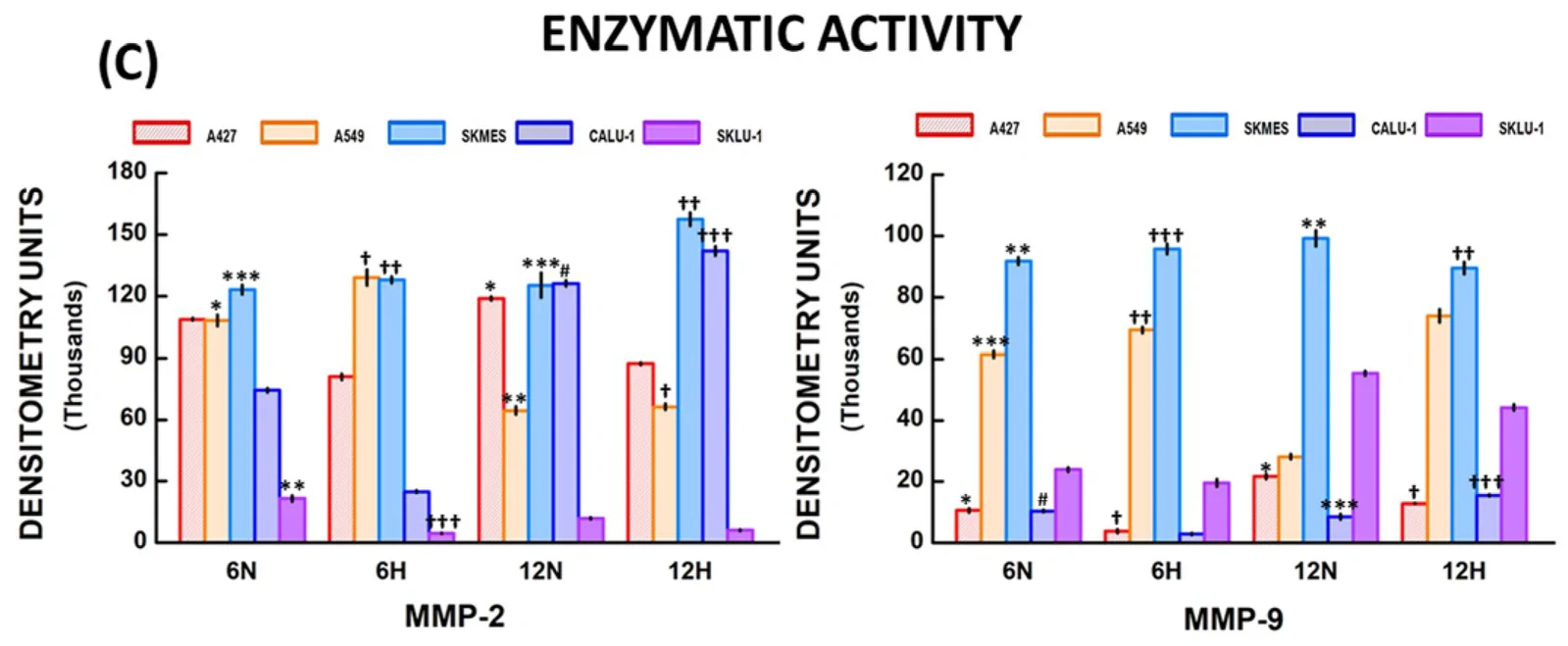
MMPs’ enzymatic activity in lung cancer cells exposed to hypoxic conditions: (**A**) Zymography assay revealed the presence of lytic bands that correspond to MMP-2 and MMP-9 enzymatic activity in all NSCLC; (**B**) The densitometry analysis demonstrated differences in the MMPs’ enzymatic activity between normoxic (N) and hypoxic (H) conditions in almost all cell lines; (**C**) MMP-2 and MMP-9 enzymatic activity was significantly different among cell lines in normoxia (N) and hypoxia (H) at 6 and 12 h. Bars represent mean ± standard error of the mean of 6 zymograms. Significant differences between cell lines are indicated by * *p*, ** *p*, *** *p*, ^†^ *p*, ^††^ *p*, ^†††^ *p*, ^‡^ *p,* ^#^ *p*; for further explanation refer to Section 2.3.6. “MMPs’ enzymatic activity among NSCLC cells”.Total protein quantification was used to normalize zymograms. Abbreviations: HT = HT-1080 sarcoma cells, MMP = matrix metalloproteinase, NSCLC = non-small cell lung cancer.

**Figure 4:**
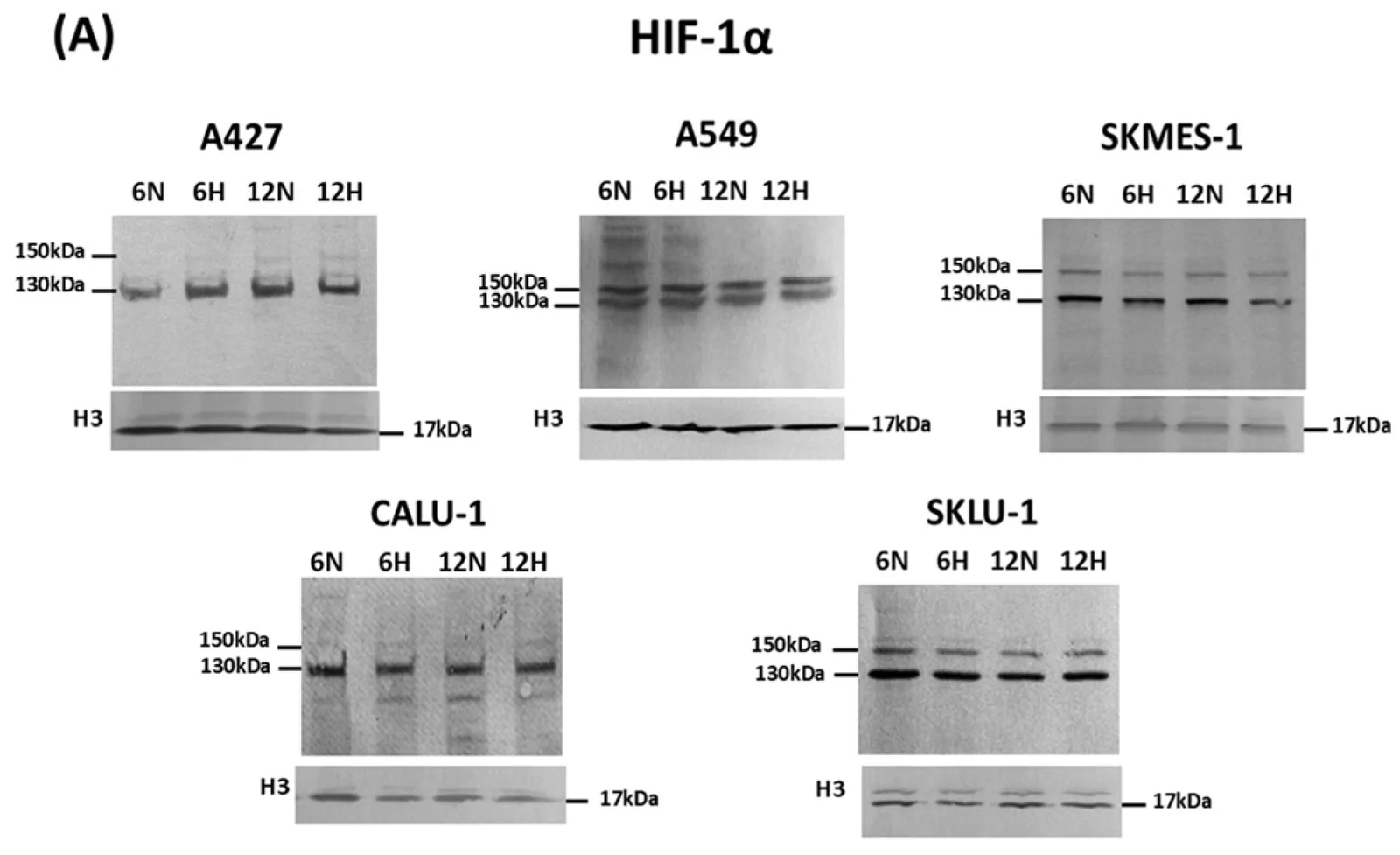

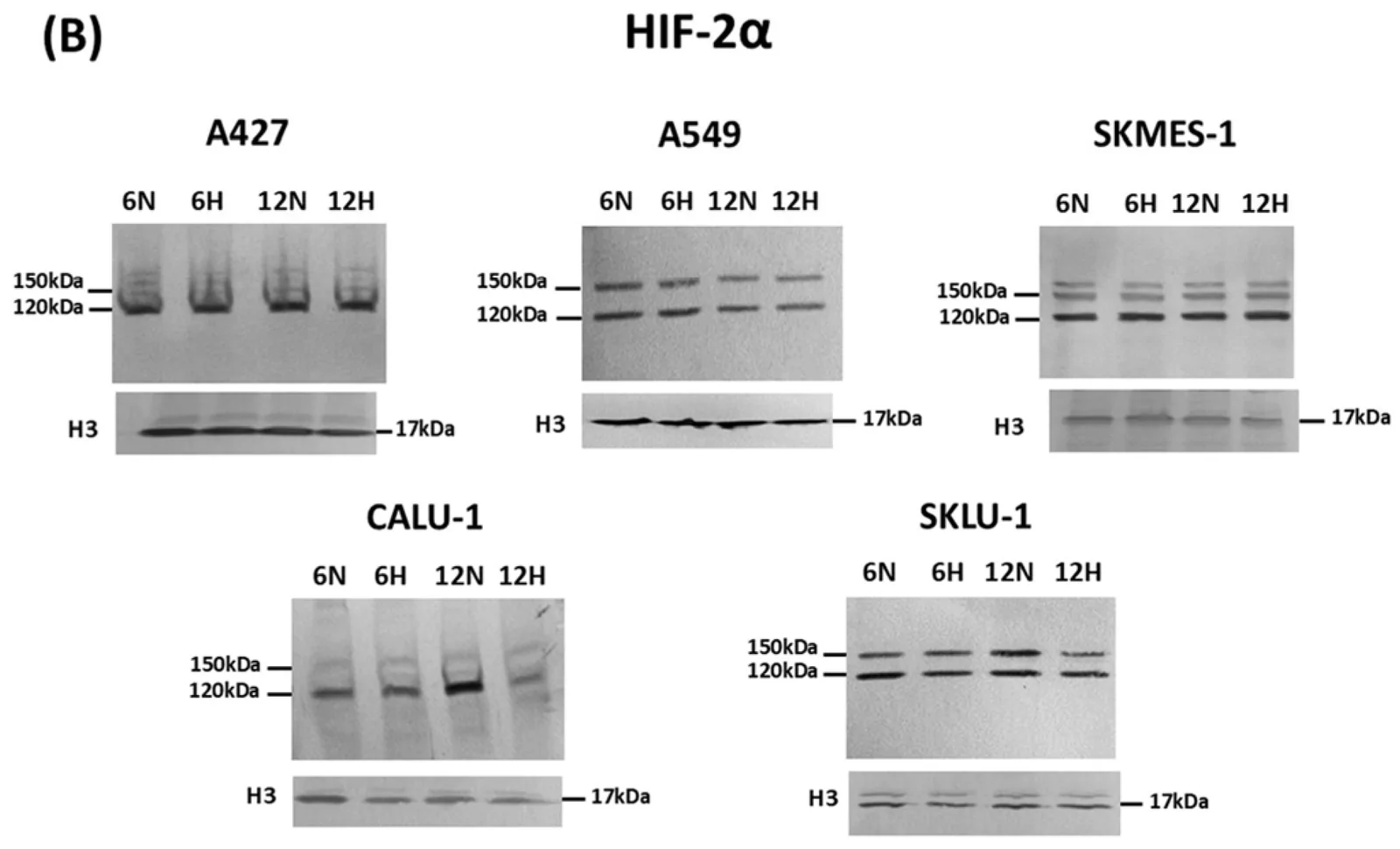

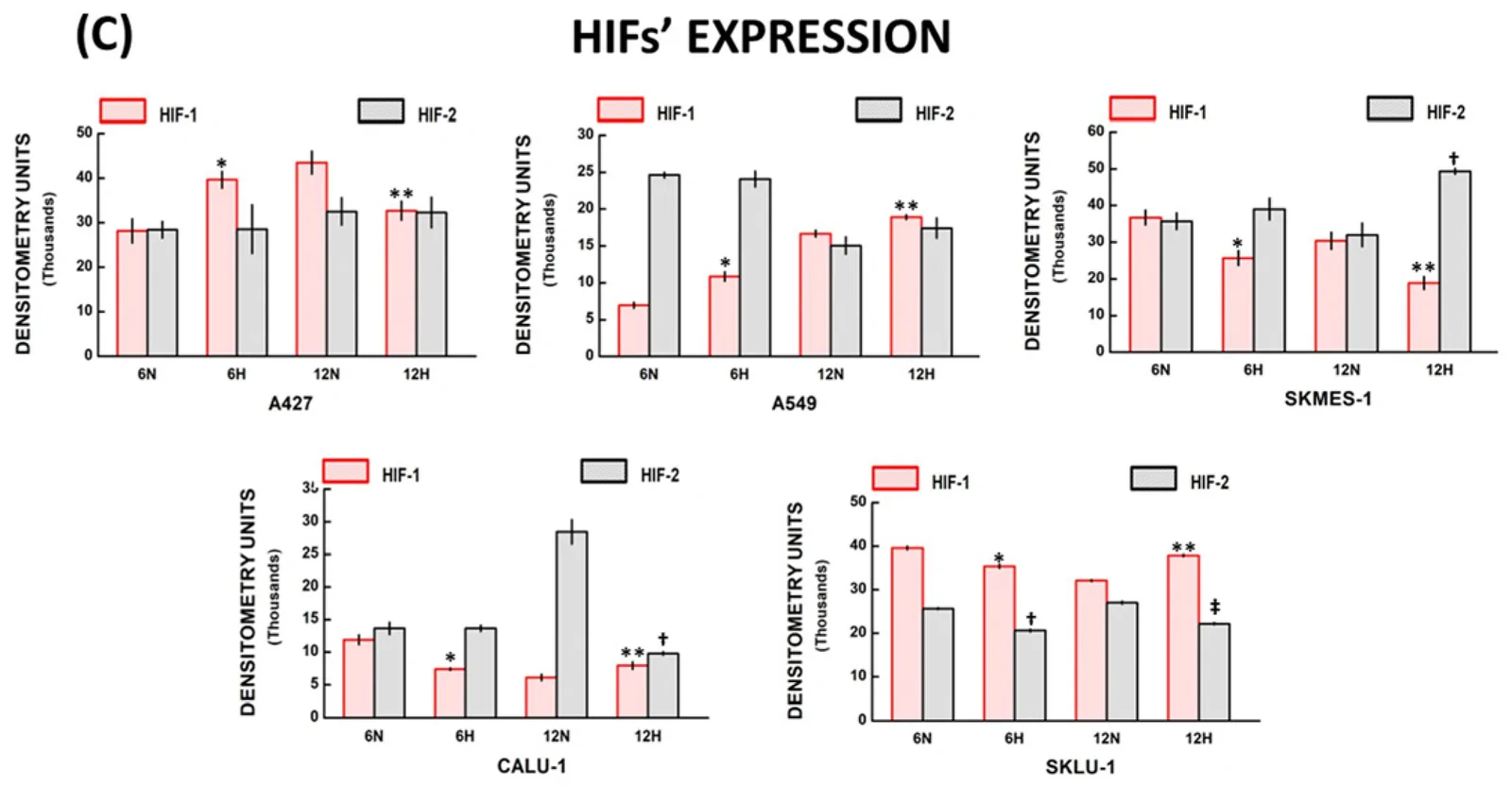

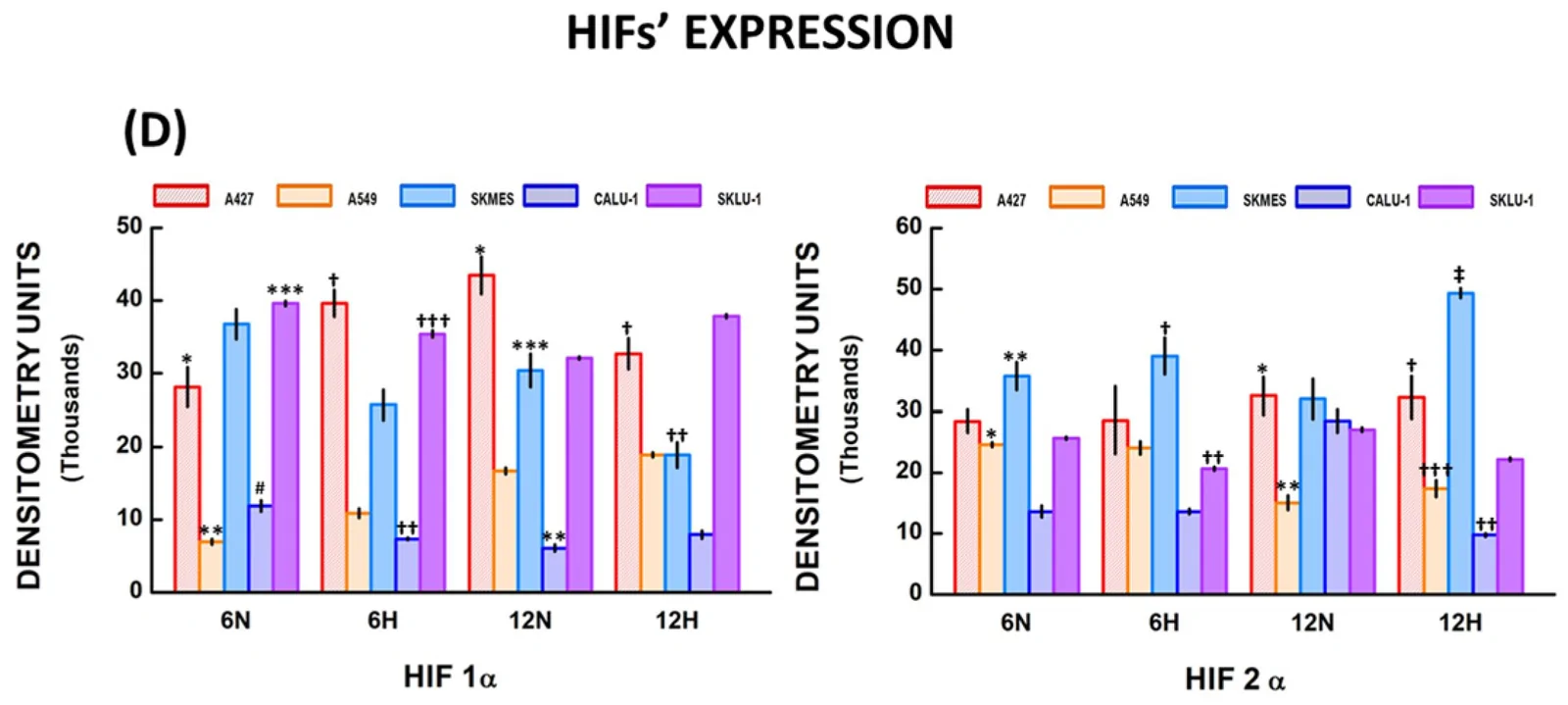
HIFs’ expression in lung cancer cells. (**A**) HIF-1α (130 kDa) and HIF-1α dimers (150 kDa) were detected by Western blot in all cell lines; (**B**) HIF-2α (120 kDa) and HIF-2α dimers (150 kDa) were observed in almost all lung cancer cell; (**C**) Densitometry analysis showed differences between HIFs protein expression in cells grown under normoxic (N) or hypoxic (H) conditions at 6 and 12 h; (**D**) Significant differences were detected in HIF-1α and HIF-2α expression among NSCLC cells in normoxic (N) and hypoxic (H) conditions. Bars represent mean ± standard error of the mean of 6 Western blots. Significant differences between cell lines are indicated by * *p*, ** *p*, *** *p*, ^†^ *p*, ^††^ *p*, ^†††^ *p*, ^‡^ *p,* ^#^ *p*; for further explanation refer to Section 2.4.6. “HIFs’ protein expression among NSCLC cells”. Abbreviations: Hypoxia-inducible factor (HIF), NSCLC = non-small cell lung cancer.

**Figure 5:**
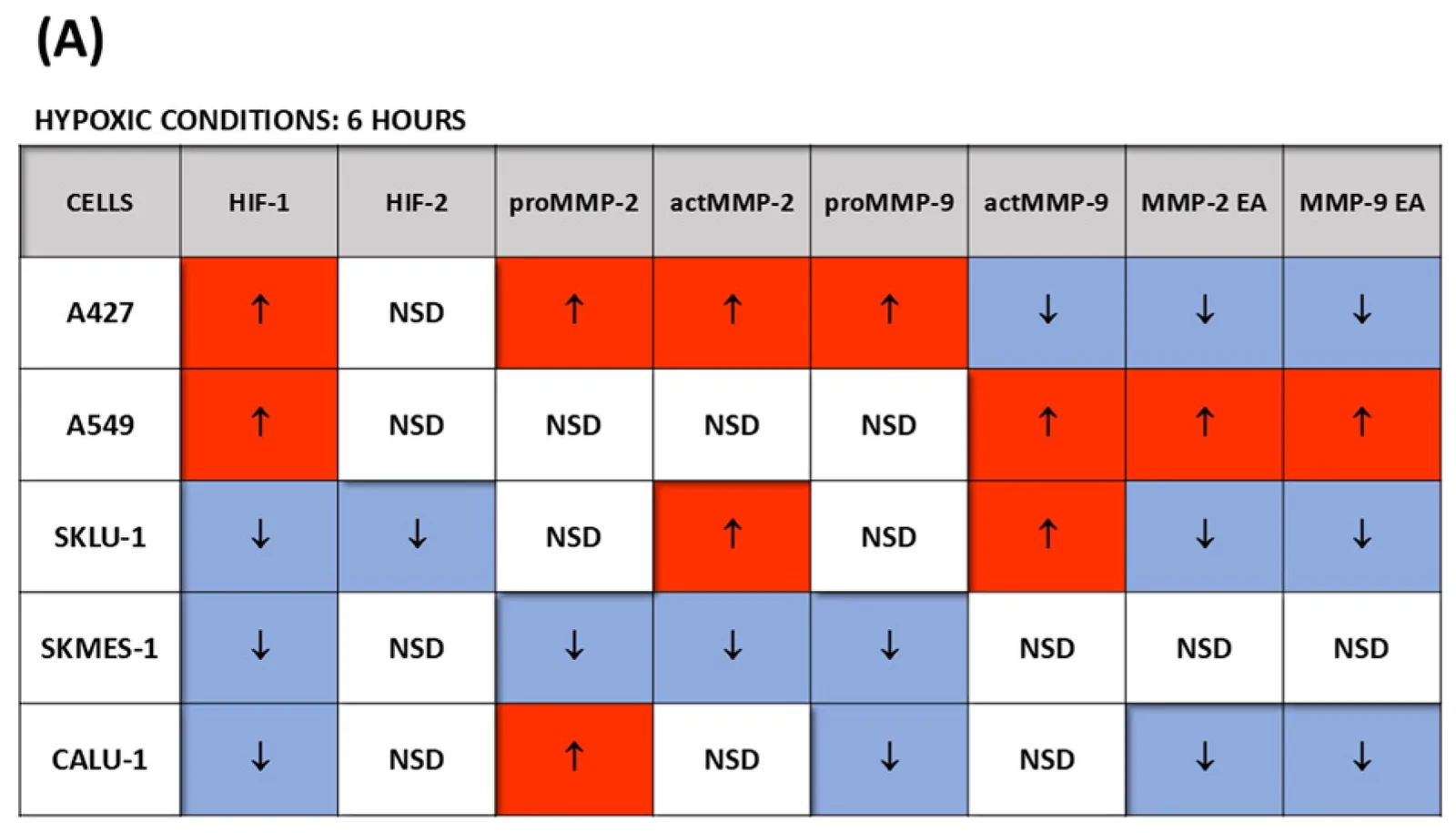

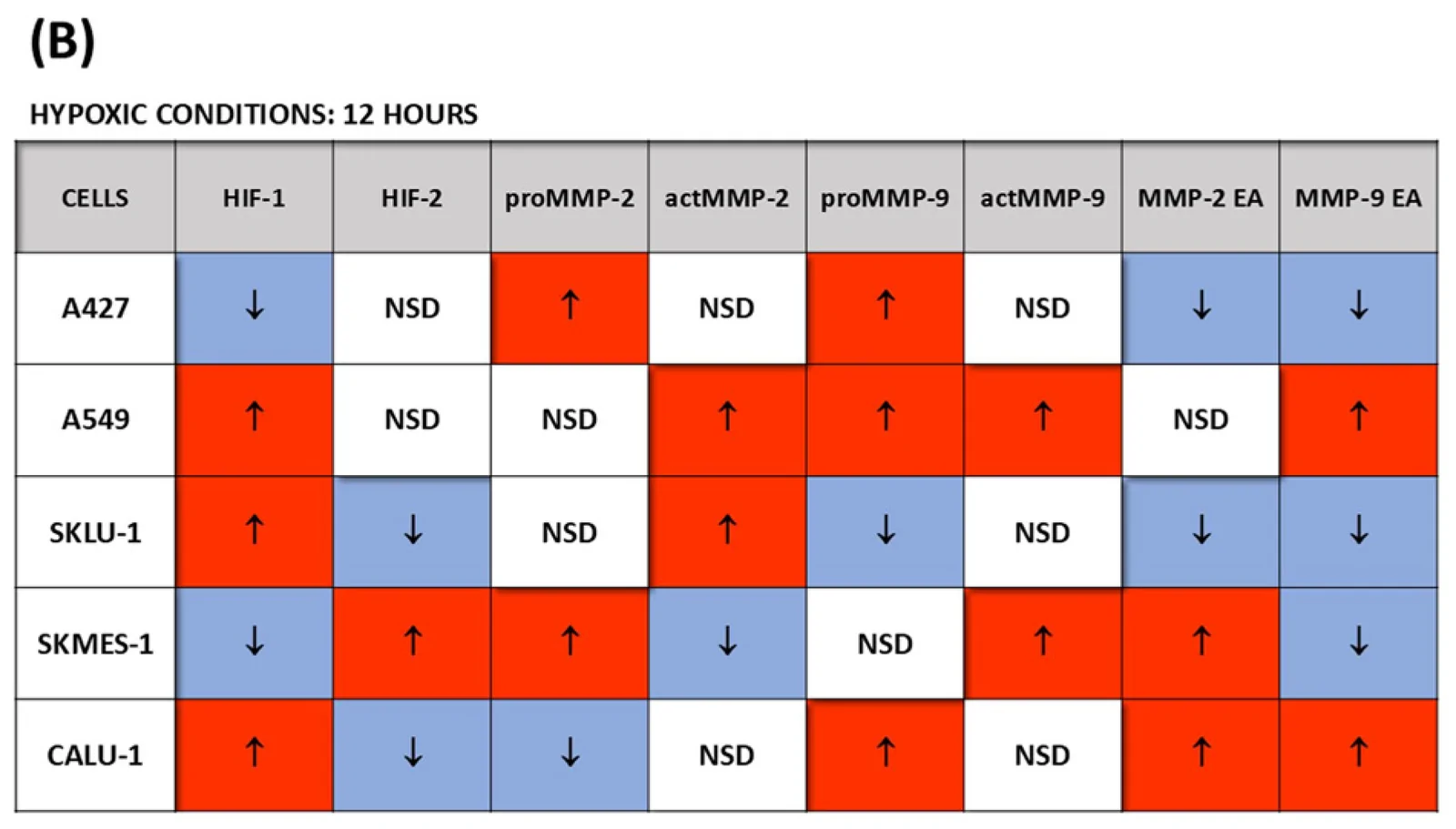
MMPs’ and HIFs’ expression in NSCLC cells grown in hypoxic conditions for 6 or 12 h. The arrows indicate increases (red color) or decreases (blue color) in the cells’ responses (either in protein expression or enzymatic activity) after hypoxic exposure relative to normoxic conditions. (**A**) HIFs’ protein levels and MMPs’ protein expression and enzymatic activity under hypoxia compared with normoxic conditions at 6 h; (**B**) HIFs’ protein levels and MMPs’ protein expression and enzymatic activity after exposure to hypoxia for 12 h in comparison with normoxia. EA = enzymatic activity; MMP = matrix metalloproteinase; NSCLC = non-small cell lung cancer; NSD = no significant differences.

## Data Availability

The original contributions presented in this study are included in the article/Supplementary Material. Further inquiries can be directed to the corresponding author.

## Supplementary Materials

The following supporting information can be downloaded at: https://www.mdpi.com/article/10.3390/ijms27209314/s1.

## Abbreviations

The following abbreviations are used in this manuscript:

GPI	Glycosylphosphatidylinositol
ECN	Eurycomanone
EPAS1	Endothelial PAS domain-containing protein 1
HIFs	Hypoxia-inducible factors
HRE	Hypoxia response element
IARC	International Agency for Research on Cancer
ITH	Intratumor heterogeneity
MMPs	Matrix metalloproteinases
NSCLC	Non-small cell lung cancer
pVHL	von Hippel–Lindau protein
SCLC	Small cell lung cancer
TME	Tumor microenvironment
TNM	Tumor Node Metastasis

## References

[1] Global Cancer Observatory. Cancer Today/IARC. https://gco.iarc.who.int.

[2] Dillekås H., Rogers M.S., Straume O. (2019). Are 90% of Deaths from Cancer Caused by Metastases?. Cancer Med..

[3] Martincorena I., Raine K.M., Gerstung M., Dawson K.J., Haase K., Van Loo P., Davies H., Stratton M.R., Campbell P.J. (2017). Universal Patterns of Selection in Cancer and Somatic Tissues. Cell.

[4] Corrales L., Rosell R., Cardona A.F., Martín C., Zatarain-Barrón Z.L., Arrieta O. (2020). Lung cancer in never smokers: The role of different risk factors other than tobacco smoking. Crit. Rev. Oncol. Hematol..

[5] Zhang A., Miao K., Sun H., Deng C.X. (2022). Tumor Heterogeneity Reshapes the Tumor Microenvironment to Influence Drug Resistance. Int. J. Biol. Sci..

[6] Fares J., Fares M.Y., Khachfe H.H., Salhab H.A., Fares Y. (2020). Molecular Principles of Metastasis: A Hallmark of Cancer Revisited. Signal Transduct. Target Ther..

[7] Gonzalez-Avila G., Sommer B., Mendoza-Posada D.A., Ramos C., Garcia-Hernandez A.A., Falfan-Valencia R. (2019). Matrix Metalloproteinases Participation in the Metastatic Process and their Diagnostic and Therapeutic Applications in Cancer. Crit. Rev. Oncol. Hematol..

[8] Rankin E.B., Giaccia A.J. (2016). Hypoxic Control of Metastasis. Science.

[9] Dengler V.L., Galbraith M., Espinosa J.M. (2014). Transcriptional Regulation by Hypoxia Inducible Factors. Crit. Rev. Biochem. Mol. Biol..

[10] Li M., Yan T., Cai Y., Wei Y., Xie Q. (2023). Expression of Matrix Metalloproteinases and their Association with Clinical Characteristics of Solid Tumors. Gene.

[11] Piperigkou Z., Mangani S., Kremmydas S., Koletsis N.E., Karamanos N.K. (2026). A guide to the types, structures, and multifaceted functions of matrix metalloproteinases in cancer. FEBS J..

[12] Sun J., Zhang Z., Xia B., Yao T., Ge F., Yan F. (2025). Overexpression of PIK3CG in Cancer Cells Promotes Lung Cancer Cell Migration and Metastasis through Enhanced MMPs Expression and Neutrophil Recruitment and Activation. Biochem. Genet..

[13] Wang Q., Zhang X.Q., Liu S.S., Liu X.Y., Lv X.J., Zhang L., Lv H. (2025). Role of GNL3L in Lung Cancer: Mediating Proliferation and Progression through NF-κB Pathway Activation and Upregulation of SLUG, MMP-2 and MMP-9. Exp. Cell Res..

[14] Soddaen P., Chairatvit K., Pitchakarn P., Laowanitwattana T., Imsumran A., Wongnoppavich A. (2025). Eurycomanone Blocks TGF-β1-Induced Epithelial-to-Mesenchymal Transition, Migration, and Invasion Pathways in Human Non-Small Cell Lung Cancer Cells by Targeting Smad and Non-Smad Signaling. Int. J. Mol. Sci..

[15] Lv Y., Wang L., Zhang Y., Wei D., Hu Y. (2025). circDENND4C Serves as a Sponge for miR-200b to Drive Non-Small Cell Lung Cancer Advancement by Regulating MMP-9 Expression. Front. Oncol..

[16] Zito Marino F., Bianco R., Accardo M., Ronchi A., Cozzolino I., Morgillo F., Rossi G., Franco R. (2019). Molecular Heterogeneity in Lung Cancer: From Mechanisms of Origin to Clinical Implications. Int. J. Med. Sci..

[17] Ashouri A., Zhang C., Gaiti F. (2023). Decoding Cancer Evolution: Integrating Genetic and Non-Genetic Insights. Genes.

[18] Wang L., Feng Y., Liu Q. (2026). Genomic instability, postoperative recurrence and therapeutic vulnerabilities in resectable non-small cell lung cancer (Review). Oncol. Rep..

[19] Iniesta P., Morán A., De Juan C., Gómez A., Hernando F., García-Aranda C., Frías C., Díaz-López A., Rodríguez-Jiménez F.J., Balibrea J.L. (2007). Biological and Clinical Significance of MMP-2, MMP-9, TIMP-1 and TIMP-2 in Non-Small Cell Lung Cancer. Oncol. Rep..

[20] Guo C.B., Wang S., Deng C., Zhang D.L., Wang F.L., Jin X.Q. (2007). Relationship between Matrix Metalloproteinase 2 and Lung Cancer Progression. Mol. Diagn. Ther..

[21] Zhang L., Li N., Yan H.C., Jiang H., Fang X.J. (2017). Expression of Novel CD44st and MMP-2 in NSCLC Tissue and their Clinical Significance. Oncol. Res. Treat..

[22] Han L., Sheng B., Zeng Q., Yao W., Jiang Q. (2020). Correlation between MMP2 expression in lung cancer tissues and clinical parameters: A retrospective clinical analysis. BMC Pulm. Med..

[23] Alonso A., de la Gala F., Vara E., Hortal J., Piñeiro P., Reyes A., Simón C., Garutti I. (2024). Lung and blood perioperative metalloproteinases in patients undergoing oncologic lung surgery: Prognostic implications. Thorac. Cancer.

[24] Liu C.H., Di Y.P. (2023). Matrix Metallopeptidase-Gene Signature Predicts Stage I Lung Adenocarcinoma Survival Outcomes. Int. J. Mol. Sci..

[25] Zhang T.Q., Lv Q.Y., Jin W.L. (2024). The Cellular-Centered View of Hypoxia Tumor Microenvironment: Molecular Mechanisms and Therapeutic Interventions. Biochim. Biophys. Acta Rev. Cancer.

[26] Zhang J., Yao M., Xia S., Zeng F., Liu Q. (2025). Systematic and Comprehensive Insights into HIF-1 Stabilization under Normoxic Conditions: Implications for Cellular Adaptation and Therapeutic Strategies in Cancer. Cell. Mol. Biol. Lett..

[27] Lee C.H., Lee M.K., Kang C.D., Kim Y.D., Park D.Y., Kim J.Y., Sol M.Y., Suh K.S. (2003). Differential Expression of Hypoxia Inducible Factor-1 Alpha and Tumor Cell Proliferation Between Squamous Cell Carcinomas and Adenocarcinomas among Operable Non-Small Cell Lung Carcinomas. J. Korean Med. Sci..

[28] Swinson D.E., Jones J.L., Cox G., Richardson D., Harris A.L., O’Byrne K.J. (2004). Hypoxia-Inducible Factor-1 Alpha in Non Small Cell Lung Cancer: Relation to Growth Factor, Protease and Apoptosis Pathways. Int. J. Cancer.

[29] Kim S.J., Rabbani Z.N., Dewhirst M.W., Vujaskovic Z., Vollmer R.T., Schreiber E.G., Oosterwijk E., Kelley M.J. (2005). Expression of HIF-1 Alpha, CA IX, VEGF, and MMP-9 in Surgically Resected Non-Small Cell Lung Cancer. Lung Cancer.

[30] Ren W., Mi D., Yang K., Cao N., Tian J., Li Z., Ma B. (2013). The Expression of Hypoxia-Inducible Factor-1α and its Clinical Significance in Lung Cancer: A Systematic Review and Meta-Analysis. Swiss Med. Wkly..

[31] Yang S.L., Ren Q.G., Wen L., Hu J.L. (2016). Clinicopathological and Prognostic Significance of Hypoxia-Inducible Factor-1 Alpha in Lung Cancer: A Systematic Review with Meta-Analysis. J. Huazhong Univ. Sci. Technol. Med. Sci..

[32] Saxena K., Jolly M.K. (2019). Acute vs. Chronic vs. Cyclic Hypoxia: Their Differential Dynamics, Molecular Mechanisms, and Effects on Tumor Progression. Biomolecules.

[33] Uchida T., Rossignol F., Matthay M.A., Mounier R., Couette S., Clottes E., Clerici C. (2004). Prolonged Hypoxia Differentially Regulates Hypoxia-Inducible Factor (HIF)-1 Alpha and HIF-2alpha Expression in Lung Epithelial Cells: Implication of Natural Antisense HIF-1alpha. J. Biol. Chem..

[34] Wu X.H., Qian C., Yuan K. (2011). Correlations of Hypoxia-Inducible Factor-1α/ /Hypoxia-Inducible Factor-2α Expression with Angiogenesis Factors Expression and Prognosis in Non-Small Cell Lung Cancer. Chin. Med. J..

[35] Gao Z.J., Wang Y., Yuan W.D., Yuan J.Q., Yuan K. (2017). HIF-2α not HIF-1α Overexpression Confers Poor Prognosis in Non-Small Cell Lung Cancer. Tumour Biol..

[36] Chen H.Y., Li G.H., Tan G.C., Liang H., Lai X.H., Huang Q., Zhong J.Y. (2019). Dexmedetomidine Enhances Hypoxia-Induced Cancer Cell Progression. Exp. Ther. Med..

[37] Paneerselvam C., Ganapasam S. (2020). β-Escin Alleviates Cobalt Chloride-Induced Hypoxia-Mediated Apoptotic Resistance and Invasion Via ROS-Dependent HIF-1α/TGF-β/MMPs in A549 Cells. Toxicol. Res..

[38] Shen J., Han L., Xue Y., Li C., Jia H., Zhu K. (2022). Ropivacaine Inhibits Lung Cancer Cell Malignancy Through Downregulation of Cellular Signaling Including HIF-1α. In Vitr. Front. Pharmacol..

[39] Gonzalez-Avila G., Sommer B., Flores-Soto E., Aquino-Galvez A. (2023). Hypoxic Effects on Matrix Metalloproteinases’ Expression in the Tumor Microenvironment and Therapeutic Perspectives. Int. J. Mol. Sci..

[40] Zhu M., Yang M., Yang Q., Liu W., Geng H., Pan L., Wang L., Ge R., Ji L., Cui S. (2020). Chronic Hypoxia-Induced Microvessel Proliferation and Basal Membrane Degradation in the Bone Marrow of Rats Regulated Through the IL6/JAK2/STAT3/MMP-9 Pathway. Biomed. Res. Int..

[41] An S.M., Lei H.M., Ding X.P., Sun F., Zhang C., Tang Y.B., Chen H.Z., Shen Y., Zhu L. (2017). Interleukin-6 identified as an important factor in hypoxia- and aldehyde dehydrogenase-based gefitinib adaptive resistance in non-small cell lung cancer cells. Oncol. Lett..

[42] Kogita A., Togashi Y., Hayashi H., Sogabe S., Terashima M., De Velasco M.A., Sakai K., Fujita Y., Tomida S., Takeyama Y. (2014). Hypoxia induces resistance to ALK inhibitors in the H3122 non-small cell lung cancer cell line with an ALK rearrangement via epithelial-mesenchymal transition. Int. J. Oncol..

[43] Yan Z., Zhou Y., Yang Y., Zeng C., Li P., Tian H., Tang X., Zhang G. (2022). Zinc Finger Protein 384 Enhances Colorectal Cancer Metastasis by Upregulating MMP2. Oncol. Rep..

[44] Petrella B.L., Lohi J., Brinckerhoff C.E. (2005). Identification of Membrane Type-1 Matrix Metalloproteinase as a Target of Hypoxia-Inducible Factor-2 Alpha in von Hipplel-Lindau Renal Cell Carcinoma. Oncogene.

[45] Smith P.K., Krohn R.I., Hermanson G.T., Mallia A.K., Gartner F.H., Provenzano M.D., Fujimoto E.K., Goeke N.M., Olson B.J., Klenk D.C. (1985). Measurement of Protein Using Bicinchoninic Acid. Anal. Biochem..

