# Supplementary material for: Hypoxic Microenvironment Effects on Matrix Metalloproteinases’ Expression and Enzymatic Activity, and HIFs’ Expression in Non-Small Cell Lung Cancer Cells
Source: Int J Mol Sci. 2026 Oct 20;27(20):9314. doi: 10.3390/ijms27209314 (PMC13705482; doi:10.3390/ijms27209314)
Supplement: Supplementary file 1 [file ijms-27-09314-s001.zip › Table S1 MMP-2 protein expression and enzymatic activity over time.pdf]

**Table S1.** MMP-2 protein expression and enzymatic activity over time.

| | Normoxic conditions | | | Hypoxic conditions | | |
|---|---|---|---|---|---|---|
| | **proMMP-2** | | | | | |
| **Cell lines** | **6N** | **12N** | **p** | **6H** | **12H** | **p** |
| **A427** | 19,596.6 ± 744.8 | 19,411.4 ± 580.4 | 0.451 | 26,650.4 ± 768.5 | 28,429.7 ± 1,050.2 | 0.127 |
| **A549** | 38,779.3 ± 6,329.3 | 32,675.8 ± 4,393.7 | 0.187 | 51,200.3 ± 8,731.2 | 38,007.7 ± 6,591.9 | 0.167 |
| **SKMES-1** | 8,032.6 ± 228.5 | 2,268.5 ± 267.6 | 0.001 | 2,790.1 ± 186.8 | 3,271.5 ± 187.9 | 0.087 |
| **CALU-1** | 57,982.3 ± 2,343.9 | 83,485.9 ± 2,263.4 | 0.014 | 66,935.5 ± 4,043.9 | 58,044.5 ± 3,712.6 | 0.123 |
| **SKLU-1** | 20,321.5 ± 876.1 | 16,732.6 ± 2,051.5 | 0.097 | 18,433.1 ± 1,121.1 | 16,282.6 ± 2,436.3 | 0.333 |
| | **Active MMP-2** | | | | | |
| **Cell lines** | **6N** | **12N** | **p** | **6H** | **12H** | **p** |
| **A427** | 13,643.9 ± 961.4 | 13,696.3 ± 1,098.1 | 0.458 | 21,237.8 ± 936.7 | 15,970.8 ± 592.4 | 0.028 |
| **A549** | 35,255.7 ± 8,506.4 | 25,135.6 ± 6,032.4 | 0.052 | 43,200.8 ± 7,493.7 | 103,196.4 ± 10,430.6 | 0.0003 |
| **SKMES-1** | 35,575.2 ± 752.2 | 26,371.8 ± 885.1 | 0.001 | 25,258.1 ± 1,399.5 | 22,902.7 ± 359.6 | 0.087 |
| **CALU-1** | 42,165.1 ± 2,000.7 | 41,910.9 ± 190.4 | 0.331 | 44,746.8 ± 1,315.2 | 43,165.4 ± 695.5 | 0.331 |
| **SKLU-1** | 32,492.1 ± 917.9 | 34,380.8 ± 2,116.2 | 0.419 | 49,137.4 ± 3,632.2 | 46,038.9 ± 4,851.8 | 0.17 |
| | **MMP-2 Enzymatic activity** | | | | | |
| **Cell lines** | **6N** | **12N** | **p** | **6H** | **12H** | **p** |
| **A427** | 108,700.5 ± 611.1 | 118,804.8 ± 855.9 | 0.003 | 81,017.8 ± 1,453.3 | 87,398.6 ± 586.5 | 0.007 |
| **A549** | 108,150.2 ± 2,786.5 | 64,549.1 ± 1,873.8 | 0.003 | 129,138.4 ± 3,820.6 | 66,362.3 ± 1,443.3 | 0.003 |
| **SKMES-1** | 123,176.5 ± 2,240.3 | 125,224.7 ± 6,029.9 | 0.405 | 128,025.9 ± 1,609.9 | 157,553.9 ± 3,208.8 | 0.001 |
| **CALU-1** | 74,440.7 ± 1,018.6 | 126,230.3 ± 1,419.1 | 0.001 | 24,952.2 ± 594.5 | 142,058.5 ± 2,274.5 | 0.001 |
| **SKLU-1** | 21,597.9 ± 1,466.7 | 11,810.6 ± 464.1 | 0.001 | 4,545.8 ± 253.4 | 6,110.9 ± 270.5 | 0.002 |

Table S1 shows a comparison between 6 and 12 h in normoxic (first versus second column) and 6 and 12 h in hypoxic (fourth versus fifth column) conditions. Significant p-values in red indicate an increase, while significant p-values in blue indicate a decrease between 6 and 12 h. Data are expressed in densitometry units. N = normoxic, H = hypoxic conditions.
