# Supplementary material for: Hypoxic Microenvironment Effects on Matrix Metalloproteinases’ Expression and Enzymatic Activity, and HIFs’ Expression in Non-Small Cell Lung Cancer Cells
Source: Int J Mol Sci. 2026 Oct 20;27(20):9314. doi: 10.3390/ijms27209314 (PMC13705482; doi:10.3390/ijms27209314)
Supplement: Supplementary file 1 [file ijms-27-09314-s001.zip › Table S2 MMP-9 protein expression and enzymatic activity over time.pdf]

Table S2. MMP-9 protein expression and enzymatic activity over time.

	Normoxic conditions			Hypoxic conditions		
	**proMMP-9**					
**Cell lines**	**6N**	**12N**	**p**	**6H**	**12H**	**p**
**A427**	27,594.37 ± 639.4	14,417.1 ± 911.1	0.026	60,139.5 ± 2,446.7	56,891.4 ± 3,837.8	0.331
**A549**	3,696.1 ± 480.7	4,758.9 ± 674.4	0.188	4,807.6 ± 829.5	49,104.3 ± 5,525.5	0.028
**SKMES-1**	9,974.8 ± 622.8	5,728.7 ± 351.3	0.001	7,063.2 ± 316.6	5,472.1 ± 703.8	0.085
**CALU-1**	27,088.1 ± 665.8	47,139.8 ± 1,420.8	0.008	24,085.8 ± 952.1	51,490.4 ± 1,865.6	0.008
**SKLU-1**	12,358.8 ± 1,731.7	6,601.2 ± 687.3	0.007	11,322.7 ± 1,230.5	4,703.7 ± 399.4	0.001
	**Active MMP-9**					
**Cell lines**	**6N**	**12N**	**p**	**6H**	**12H**	**p**
**A427**	22,071.6 ± 878.8	0	---	0	0	---
**A549**	9,123.1 ± 1,354.1	11,468.1 ± 2,445.6	0.333	16,910.4 ± 2,777.1	71,570.5 ± 7,661.3	0.003
**SKMES-1**	8,089.6 ± 537.5	8,480.9 ± 784.1	0.389	8,178.4 ± 618.1	13,025.1 ± 912.3	0.01
**CALU-1**	49,993.8 ± 1,955.6	52,186.3 ± 4,967.6	0.464	50,518.3 ± 3,645.4	54,467.3 ± 3,861.6	0.206
**SKLU-1**	35,790.3 ± 1,149.1	32,621.6 ± 1,145.4	0.05	44,455.1 ± 1,250.9	31,608.4 ± 1,268.9	0.001
	**MMP-9 Enzymatic activity**					
**Cell lines**	**6N**	**12N**	**p**	**6H**	**12H**	**p**
**A427**	10,599.6 ± 655.3	21,675.4 ± 863.7	0.003	3,813.2 ± 601.3	12,815.7 ± 76.4	0.003
**A549**	61,517.5 ± 1,080.4	28,150.6 ± 821.6	0.003	69,290.4 ± 1,036.6	73,967.8 ± 2,132.8	0.05
**SKMES-1**	91,831.6 ± 1,168.8	99,315.3 ± 2,569.3	0.05	95,749.4 ± 1,667.9	89,584.2 ± 1,963.8	0.05
**CALU-1**	10,435.1 ± 368.1	8,525.4 ± 970.1	0.115	2,991.6 ± 292.8	15,497.4 ± 300.4	0.003
**SKLU-1**	23,988.5 ± 659.9	55,360.4 ± 774.3	0.001	19,553.6 ± 1,196.1	44,217.8 ± 976.1	0.001

Table S2 shows a comparison between 6 and 12 h in normoxic (first versus second column) and 6 and 12 h in hypoxic (fourth versus fifth column) conditions. Significant p-values in red indicate an increase, while significant p-values in blue indicate a decrease between 6 and 12 h. Data are expressed in densitometry units. N = normoxic, H = hypoxic conditions.
