# Supplementary material for: Hypoxic Microenvironment Effects on Matrix Metalloproteinases’ Expression and Enzymatic Activity, and HIFs’ Expression in Non-Small Cell Lung Cancer Cells
Source: Int J Mol Sci. 2026 Oct 20;27(20):9314. doi: 10.3390/ijms27209314 (PMC13705482; doi:10.3390/ijms27209314)
Supplement: Supplementary file 1 [file ijms-27-09314-s001.zip › Table S3 HIFs protein expression over time.pdf]

Table S3. HIFs' protein expression over time.

	Normoxic conditions			Hypoxic conditions		
	**HIF-1α**					
**Cell lines**	**6N**	**12N**	**p**	**6H**	**12H**	**p**
**A427**	28,141.7 ± 2,714.1	43,463.7 ± 2,563.4	0.0004	39,635.9 ± 1,846.6	32,693.7 ± 2,128.7	0.03
**A549**	6,972.1 ± 393.7	16,680.4 ± 454.4	0.001	10,858.5 ±628.7	18,915.1 ± 314.1	0.004
**SKMES-1**	36,755.1 ± 2,038.6	30,426.5 ± 2,279.6	0.03	25,705.4 ± 2,040.2	18,895.1 ± 1,748.1	0.03
**CALU-1**	11,893.4 ± 739.9	6,109.1 ± 485.7	0.00007	7,377.7 ± 173.3	7,947.2 ± 556.6	0.485
**SKLU-1**	39,602.4 ± 366.2	32,121.2 ± 202.6	0.003	35,404.9 ± 460.9	37,851.6 ± 260.5	0.003
	**HIF-2α**					
**Cell lines**	**6N**	**12N**	**p**	**6H**	**12H**	**p**
**A427**	28,405.3 ± 1,850.9	32,530.5 ± 3,047.4	0.125	28,578.3 ± 5,463.1	32,287.1 ± 3,435.4	0.213
**A549**	24,619.5 ± 393	15,067.1 ± 1,168.2	0.003	24,084.9 ± 1,059.7	17,427.1 ± 1,364.7	0.004
**SKMES-1**	35,736.4 ± 2,276.6	32,033.2 ± 3,227	0.158	39,031.7 ± 2,975.6	49,345.1 ± 795.5	0.134
**CALU-1**	13,634.3 ± 918.4	28,460.1 ± 1,856.7	0.014	13,634.81 ± 453.7	9,784.68 ± 255.4	0.014
**SKLU-1**	25,664.3 ± 216.9	27,034.7 ± 346.7	0.008	20,635.4 ± 313.3	22,208.6 ± 228.2	0.004

Table S3 shows a comparison between 6 and 12 h in normoxic (first versus second column) and 6 and 12 h in hypoxic (fourth versus fifth column) conditions. Significant p-values in red indicate an increase, while significant p-values in blue indicate a decrease between 6 and 12 h. Data are expressed in densitometry units. N = normoxic, H = hypoxic conditions.
